# Machine learning for prediction of asthma exacerbations among asthmatic patients: a systematic review and meta-analysis

**DOI:** 10.1186/s12890-023-02570-w

**Published:** 2023-07-28

**Authors:** Shiqiu Xiong, Wei Chen, Xinyu Jia, Yang Jia, Chuanhe Liu

**Affiliations:** 1 Department of Allergy, Center for Asthma Prevention and Lung Function Laboratory, Children’s Hospital of Capital Institute of Pediatrics, Beijing, 100020 China; 2 Graduate School, Peking Union Medical College, Beijing, 100730 China; 3 Department of Pediatrics, The Second Xiangya Hospital, Central South University, Changsha, 410011 Hunan China

**Keywords:** Asthma, Exacerbation, Prediction model, Machine learning, Systematic review, Meta-analysis

## Abstract

**Background:**

Asthma exacerbations reduce the patient’s quality of life and are also responsible for significant disease burdens and economic costs. Machine learning (ML)-based prediction models have been increasingly developed to predict asthma exacerbations in recent years. This systematic review and meta-analysis aimed to identify the prediction performance of ML-based prediction models for asthma exacerbations and address the uncertainty of whether modern ML methods could become an alternative option to predict asthma exacerbations.

**Methods:**

PubMed, Cochrane Library, EMBASE, and Web of Science were searched for studies published up to December 15, 2022. Studies that applied ML methods to develop prediction models for asthma exacerbations among asthmatic patients older than five years and were published in English were eligible. The prediction model risk of bias assessment tool (PROBAST) was utilized to estimate the risk of bias and the applicability of included studies. Stata software (version 15.0) was used for the random effects meta-analysis of performance measures. Subgroup analyses stratified by ML methods, sample size, age groups, and outcome definitions were conducted.

**Results:**

Eleven studies, including 23 prediction models, were identified. Most of the studies were published in recent three years. Logistic regression, boosting, and random forest were the most used ML methods. The most common important predictors were systemic steroid use, short-acting beta2-agonists, emergency department visit, age, and exacerbation history. The overall pooled area under the curve of the receiver operating characteristics (AUROC) of 11 studies (23 prediction models) was 0.80 (95% CI 0.77–0.83). Subgroup analysis based on different ML models showed that boosting method achieved the best performance, with an overall pooled AUROC of 0.84 (95% CI 0.81–0.87).

**Conclusion:**

This study identified that ML was the potential tool to achieve great performance in predicting asthma exacerbations. However, the methodology within these models was heterogeneous. Future studies should focus on improving the generalization ability and practicability, thus driving the application of these models in clinical practice.

**Supplementary Information:**

The online version contains supplementary material available at 10.1186/s12890-023-02570-w.

## Background

Asthma is a chronic heterogeneous disease affecting approximately 241 million people worldwide [1]. Despite many effective medicines available, a proportion of asthmatic patients have uncontrolled asthma and asthma exacerbations [2, 3]. Asthma exacerbations are characterized by progressive deterioration of asthma-related symptoms and lung function, resulting in a poor quality of life [4, 5]. Severe asthma exacerbations are also responsible for decreased lung function, hospitalization, and even death, thus leading to disease and economic burdens [6, 7]. Early recognition and timely intervention are the best strategies to prevent severe asthma exacerbations. Therefore, identifying patients at high risk of asthma exacerbations is crucial.

According to a systematic review including ten prediction models for asthma exacerbations, the best prediction performance was achieved by logistic regression (LR) with a c-statistic of 0.80 [8]. However, this systematic review did not include models based on modern machine learning (ML) algorithms, such as random forest (RF), neural network (NN), boosting algorithms, and support vector machine (SVM). ML has become a popular method for developing prediction models in the medical field due to its ability to process complex, massive health data [9]. Many studies developing prediction models for asthma exacerbations based on ML methods have been published, especially in recent years [10, 11]. However, few systematic reviews were conducted to evaluate these existing ML models. Therefore, we perform a systematic review and meta-analysis to estimate the prediction performance of ML-based prediction models for asthma exacerbations and identify whether modern ML methods could become an alternative option to prediction.

## Methods

### Search protocol

We conducted this systematic review in accordance with Preferred Reporting Items for a Systematic Review and Meta-analysis of Diagnostic Test Accuracy Studies (The PRISMA-DTA Statement). The protocol of this systematic review was registered and published on PROSPERO (reference number CRD42022380059).

### Search strategy

PubMed, Cochrane Library, EMBASE, and Web of Science were searched for relevant literature published from the earliest available online date up to December 15, 2022. Our search strategies used controlled terms and free-text terms to search for studies of the ML approach and asthma exacerbations. Details of the search strategy are given in Additional file 1. We also checked reference lists of previous systematic reviews for potentially relevant papers.

### Eligibility criteria and study selection

All search records were exported from the four databases and imported to EndNote 20 (Clarivate), a reference management tool, for compiling and duplication checking. After removing the duplications, two reviewers (SQ, X and XY, J) independently screened the titles and abstracts to select the studies based on inclusion criteria. Subsequently, they screened the full texts to select eligible studies. Any discrepancies were resolved by a third reviewer (W, C).

All studies should fulfill the criteria as follows:

a Studies must be published in English.
b Focused on participants aged five years and older with pre-existing asthma diagnoses.
c Utilized machine learning algorithms to generate prediction models.
d Aimed to predict patients who would suffer asthma exacerbations in the future.
e Evaluated the prediction performance of models on a validation dataset.
f Provided a clear description of ML methods and input features (predictors).
g Provided the performance metrics regarding sensitivity and specificity.

We did not limit the type of publication and study designs.

### Data extraction

Two reviewers (SQ, X and Y, J) independently read the full texts of eligible studies and extracted data elements, including (1) the paper source, (2) study information, (3) prediction models, (4) performance measures, (5) population characteristics, and (6) outcomes. Full details of data extraction are provided in Additional file 2. We defined asthma exacerbations in accordance with an Official American Thoracic Society/European Respiratory Society (ATS/ERS) Statement [12]. Briefly, severe asthma exacerbation should include (1) at least three days of systemic corticosteroid treatment or (2) a hospitalization/emergency department visit for asthma requiring systemic corticosteroids. Moderate asthma exacerbation should include (1) at least two days of symptoms and lung function deterioration, requiring increasing bronchodilator use, or (2) visits for asthma not requiring systemic corticosteroids intervention. Using available statistics in the manuscripts, we manually calculated parameters not reported (e.g., the number of positive cases). We also emailed the corresponding author(s) for missing data.

### Quality and bias assessment

There are no widely accepted tools for assessing the quality of machine learning-based research in medical fields. In 2019, Wolff et al. [13] developed the prediction model risk of bias assessment tool (PROBAST), which could assess the risk of bias (ROB) and the applicability of prediction model studies. For ROB assessment, PROBAST includes four domains: participants, predictors, outcomes, and analysis. Each domain contains 2 to 9 signaling questions that facilitate this domain’s ROB assessment (low, high, or unclear). The overall ROB assessment for a study is “low,” “high,” or “unclear,” based on each domain’s ROB classification. The first three domains with review questions are also used for applicability judgment (low, high, or unclear concern). This paper used the PROBAST to assess the ROB and applicability of included studies. Two authors (SQ, X and CH, L) independently assessed eligible studies, and any disagreements were resolved by discussion.

### Data analysis

We narratively described these included studies, such as distribution of publication year, population characteristics, popular machine learning methods, validation methods, and important features. For studies that were able to calculate the number of true positive cases, true negative cases, false positive cases, and false negative cases on the validation dataset, the overall pooled area under the curve of the receiver operating characteristics (AUROC), sensitivity, specificity, positive likelihood ratio (PLR), negative likelihood ratio (NLR), and diagnostic odds ratio (DOR) were estimated using random effects meta-analysis. *I*^2^ was used to describe the percentage of the variability in effect estimates due to heterogeneity.

A small sample size causes the risk of overfitting, which may lead to poor generalization of prediction models. Subgroup analysis was stratified by sample size (< 10000 participants/ > 10000 participants). In addition, we performed a subgroup analysis of ML methods (LR, boosting, and RF), age groups (children only, children and adults, and adults only), and different outcome definitions. Univariate and multivariate random-effects meta-regression for sample size, ML methods, age groups, outcome definitions, and publication year was performed to explore the source of heterogeneity. For clarity, we referred to factors used for model development as “predictors” and the factors used for meta-regression at study level as “variables”. Sensitivity analyses were conducted to examine the robustness of the result by excluding each study. Deeks’ funnel plot was applied to test publication bias. We conducted all our analyses using Stata software (version 15.0). We used the MADIS module for pooling performance measures and the “metareg” macro for conducting the meta-regression analysis. The commands used in the analysis are provided in Additional file 2.

## Results

### Study selection

**Fig. 1:**
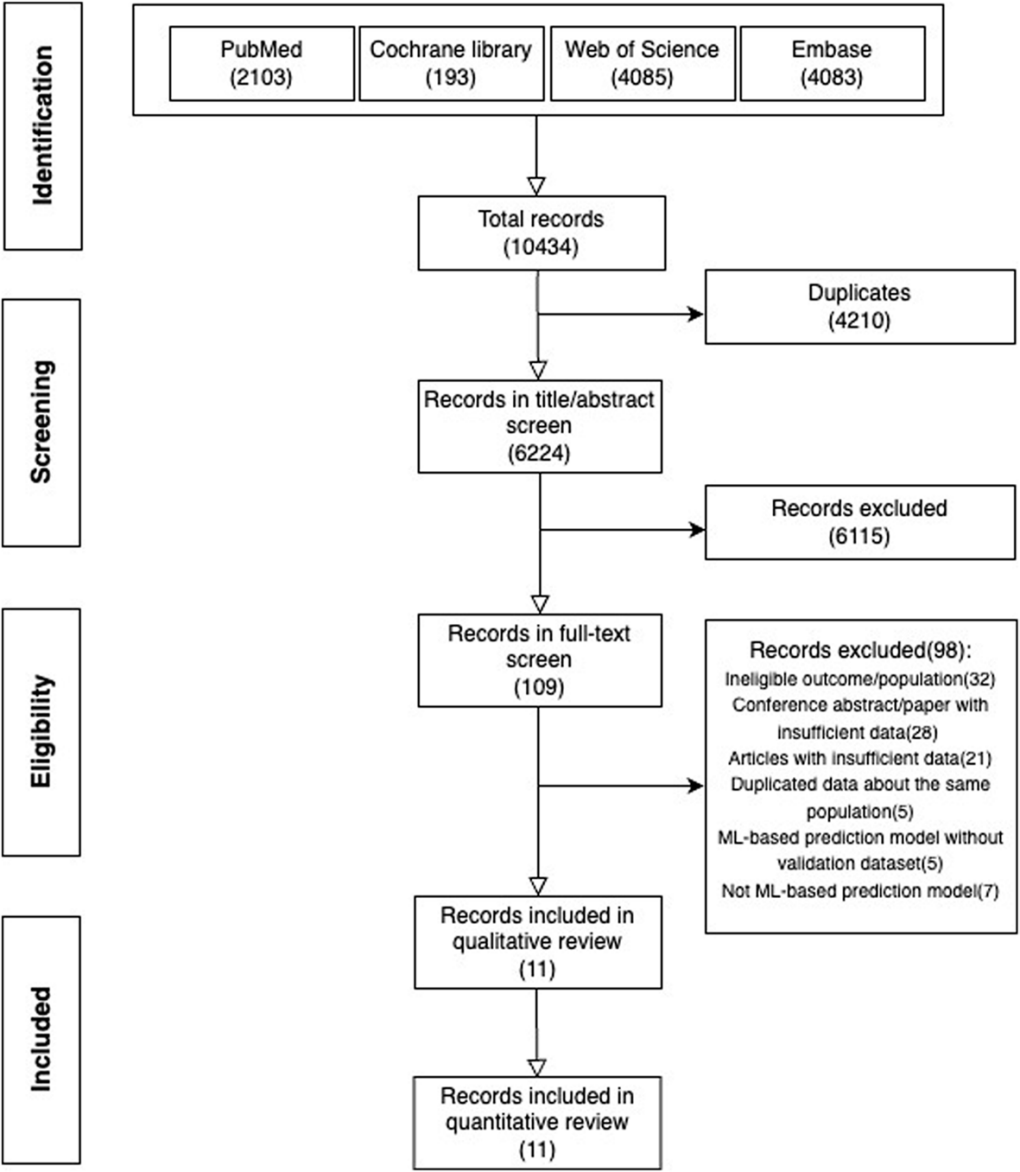
PRISMA flow diagram describing the selection process of articles

A total of 10434 papers were identified from four databases (PubMed (2013), Cochrane library (193), Web of Science (4085), Embase (4083)) (see Additional file 1). After excluding 4210 duplicates, we browsed titles and abstracts of the remaining 6224 papers resulting in 109 papers that might be eligible based on the pre-defined selection criteria. Then, we screened these papers’ full texts and supplementary materials and included 11 papers for synthesis (Fig. 1). Two studies included participants without age limitation, but only a tiny proportion of participants in these two studies were aged younger than five years old [14, 15].

### Study characteristics

The publication year of these papers ranged from 1999 to 2022, and more than half of them (6/11) were published in recent three years. Ten studies were retrospective, and the remaining one was prospective. The minimum and maximum number of included participants for prediction model development were 94 and 782762, respectively. The proportion of outcome events ranged from 0.2% to 32.8% (Table 1).

**Table 1:** Summary of included studies in this literature review

Studies (author, year)	Study design	Participants	Sample size (outcome%)	Outcome	Prediction duration	ML algorithms (n)	Validation methods	Generalization test^a^	Features in the final model	Performance measure in the validation dataset	Quality (PROBAST)	
											Bias	Applicability
Lieu, 1999 [16]	Retrospective	Asthma	7141 (HP 0.8%, ED 6.6%)	HP or ED for asthma	Next year	CART (1)	Split-sample validation	Y (in separate dataset)	14	Internal: Spe 0.836, Sen 0.49, PPV 0.185	High	High
Schatz, 2004 [17]	Retrospective	Asthma	8789 (5.5%)	HP or ED for asthma	Next year	LR (1)	-	Y (geographic)	3	External: Spe 0.92, Sen 0.254, NPV 0.932	High	High
Schatz, 2006 [18]	Retrospective	Asthma	1079 (9.5%)	HP or ED	Next year	LR (1)	-	Y (temporal)	1	External: Spe 0.541, Sen 0.646, NPV 0.96, PPV 0.083, AUC 0.59	High	High
Xu, 2011 [19]	Retrospective	Mild-moderate asthma	417 (30%)	Severe AE	Four years	RF (1)	Cross validation	Y (in separate sample)	164	External: AUC 0.66, Spe 0.6, Sen 0.66, NPV 0.81, PPV 0.74	High	High
van Vliet, 2017 [20]	Prospective	Asthma	574 samples (13.4%) from 94 patients (48%)	Moderate-severe AE	0–14 days, 0–21 days	RF (2)	Bagging	-	7	32 samples: 0–14 days (Spe 0.75, Sen 0.88, CCR 0.82, AUC 0.90) 48 samples: 0–21 days (Spe 0.67, Sen 0.85, CCR 0.65)	High	Low
Luo, 2020 [15]	Retrospective	Asthma	315,308 (3.6%)	HP or ED for asthma	Next year	XGBoost (1)	Cross validation	Y (temporal)	142	External: Spe 0.9193, Sen 0.5369, CCR 0.9031, AUC 0.859	High	High
Luo, 2020 [14]	Retrospective	Asthma	782,762 (2.42%)	HP or ED for asthma	Next year	XGBoost (1)	-	Y (temporal)	221	External: Spe 0.9091, Sen 0.5190, CCR 0.9008, AUC 0.820	High	High
Tong, 2021 [21]	Retrospective	Asthma	68,244 (1.7%)	HP or ED for asthma	Within 1 year	XGBoost (1)	Unclear	Y (temporal)	71	External: Spe 0.9091, Sen 0.7018, CCR: 0.906, AUC 0.902	High	High
Zein, 2021 [22]	Retrospective	Asthma	60,302 (nonsevere AE 32.8%, ED 2.9%, HP 1.5%)	Nonsevere AE (oral steroids burst), severe AE (HP or ED for asthma)	3 years (mean)	LR (3), RF (3), LGBM (3)	-	Y (20% of dataset, temporal)	56	20% of dataset: LGBM: Nonsevere (AUC 0.71, Spe 0.67, Sen 0.64), ED (AUC 0.88, Spe 0.76, Sen 0.84), HP (AUC 0.85, Spe 0.73, Sen 0.86))	High	High
										External: LGBM: nonsevere (AUC 0.65), ED (AUC 0.86), HP (AUC 0.87))		
Noble, 2021 [23]	Retrospective	Asthma	58,619 (1.65%)	HP for asthma	Within one year	LR (1)	-	Y (in separate dataset)	14	External: AUC 0.71, Spe 0.933, Sen 0.285	High	High
de Hond, 2022 [24]	Retrospective	Stable, mild-moderate asthma	92,787 daily measurements (0.2%) from 165 patients (30%)	Severe AE	2 days	XGBoost (1), one class SVM (1), LR (1)	Cross validation	Y (in separate dataset)	Unclear	External: XGBoost (AUC 0.81, Spe 0.89, Sen 0.59), LR: (AUC 0.88, Spe 0.82, Sen 0.84), one class SVM (Spe 0.87, Sen 0.34)	High	High

^a^Generalization tests included two types of validation. Some studies applied external validation, such as temporal and geographic validation. Some studies split a single dataset into a training dataset and a test dataset and used the latter to assess the generalizability of prediction models. We provided an example in Additional file 4

*HP* hospitalization, *ED* emergency department visit, *AE* asthma exacerbation, *Spe* specificity, *Sen* sensitivity, *PPV* positive predictive value, *NPV* negative predictive value, *AUC* area under the curve, *CCR* correct classification rate, *SVM* support vector machine, *LGBM* Light gradient boosting machine, *CART* classification and regression trees, *LR* logistic regression, *RF* random forest

Most studies (9/11) included asthmatic participants regardless of asthma severity, control levels, or treatment. Only two studies mentioned additional criteria, such as participants with mild-moderate asthma [19, 24] and stable asthma [24] (see Additional file 3). Prediction windows also varied from several days to 4 years, with seven studies setting the prediction window within one year (Table 1). For outcome events (see Additional file 3), nine studies defined asthma exacerbations as asthma-related hospitalization or emergency department visit according to the asthma-related diagnosis code [14–18, 21], medical records [22, 23], or questionnaires [19]. Two studies used the definitions in accordance with the ATS/ERS recommendation [20, 24].

### ML algorithms and validation methods

**Fig. 2:**
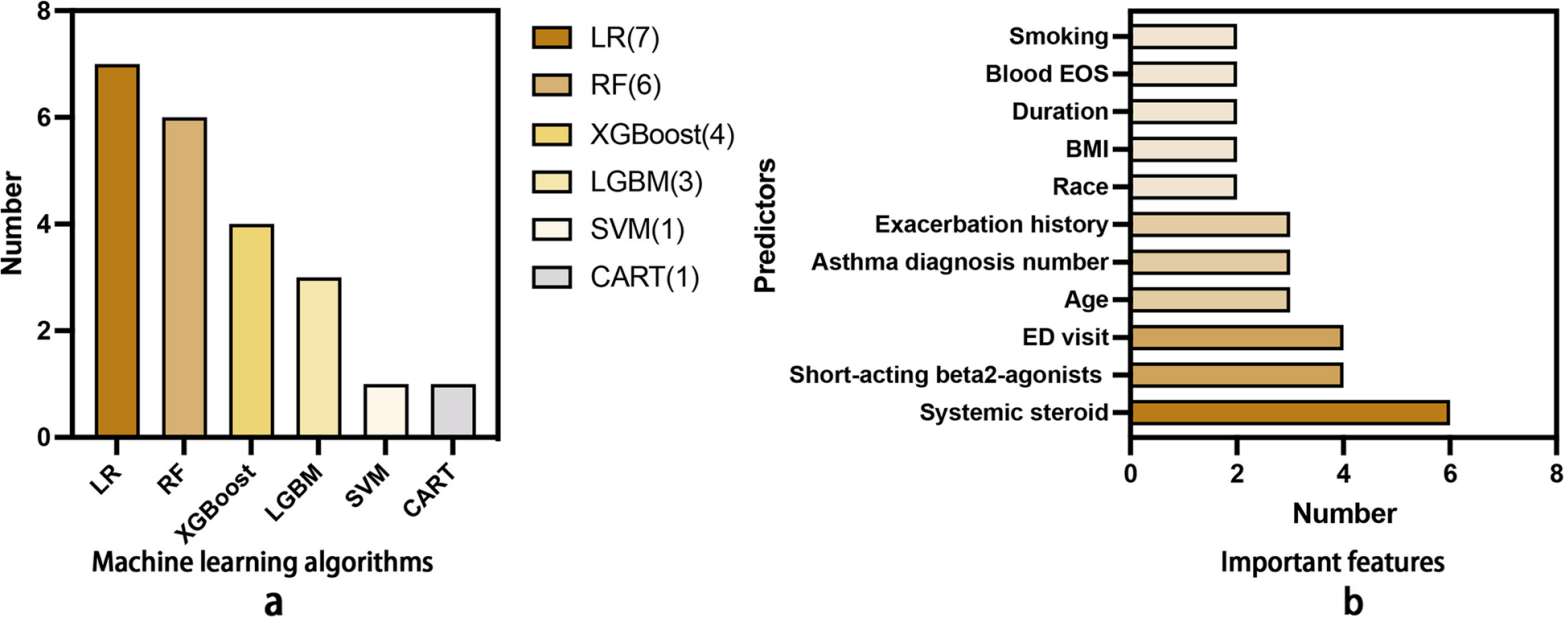
**a** Distribution of machine learning algorithms. **b** Important features among included studies

Eleven studies developed a total of 23 ML-based prediction models. The most popular ML algorithm was LR, followed by RF, XGBoost, and LGBoost (Fig. 2a). Validation methods were used in 6 studies, such as cross-validation [15, 19, 24], bagging [20], and split-sample validation [16] (Table 1). For the generalization test, ten studies used external validation. One study split a single dataset into a training dataset and a test dataset and used the latter to assess the generalization ability of prediction models. We also included more detailed descriptions of the dataset and the validation method in Additional file 4 for better clarity.

### Predictors in ML models

A wide range of predictors was used in these studies, such as demographic factors, clinical-related factors, and socioeconomic factors. Clinical-related factors (*n* = 11) and demographic factors (*n* = 7) were used most in the final models, followed by social-economic factors (*n* = 3) (see Additional file 5). The number of predictors in best prediction models ranged from 1 to 221. Most studies that applied LR and classification and regression trees (CART) to develop prediction models had a relatively minor number of predictors. The number of predictors in models based on boosting and RF was much higher (Table 1). All studies reported the predictors' contributions or odds ratios (only in LR). Among these important predictors, systemic steroids use, short-acting beta2-agonists, and emergency department visit were the most common predictors, followed by age, asthma diagnosis number, and exacerbation history (Fig. 2b, Additional file 5).

### Risk of bias and applicability

The overall quality assessment (ROB and applicability) based on PROBAST is shown in Table 1. Additional file 6 provides judgment details of each study. The overall bias of all studies was rated as high risk. For participants, eight studies were at high ROB mainly due to retrospective design and asthma definition that was based on asthma-related medicine use and doctors’ diagnosis. The bias of predictors mainly results from subjective predictors (such as self-report symptoms), auxiliary examinations from different medical institutions, and comorbidities. These factors were difficult to be defined consistently. The definition of asthma exacerbations given by the ATS/ERS statement is widely accepted [12]. Studies in which the outcome was not in accordance with ATS/ERS statement were rated as high risk of bias. All studies had a high risk of bias in the “analysis” domain.

For applicability assessment, one study was judged as low concerns, and the remains were rated as high concerns. Two studies included asthmatic participants with mild to moderate asthma [19, 24], thus might reduce the generalizability and applicability. Six studies were assessed as having high concerns in the “predictors” domain. The applicability would reduce when predictors were challenging to be defined similarly. As for the outcome, studies (10/11) would receive a rating of high concern if they did not focus on moderate to severe asthma exacerbations defined by the ATS/ERS statement.

### Meta-analysis

The discrimination ability of ML-based models was various. AUROC was reported in 21 models, the best prediction performance of asthma exacerbations ranged from 0.59 to 0.90. The specificity and sensitivity based on different cut-off points were reported in all included studies, with the range of 0.54–0.93 and 0.25–0.88, respectively. Negative predictive value (*n* = 4), positive predictive value (*n* = 4), and accuracy (*n* = 4) of prediction models in several studies were also reported (Table 1).

**Fig. 3:**
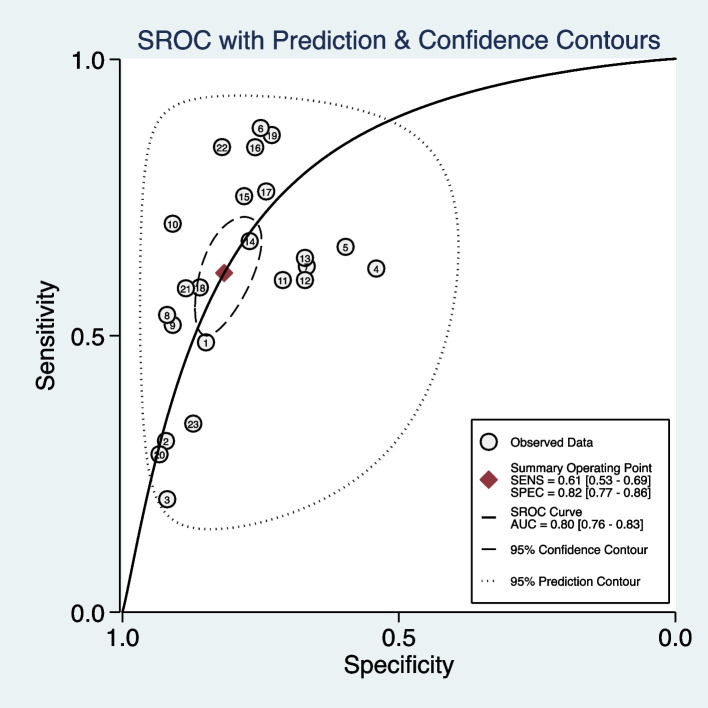
The overall pooled AUROC of machine learning prediction models

**Fig. 4:**
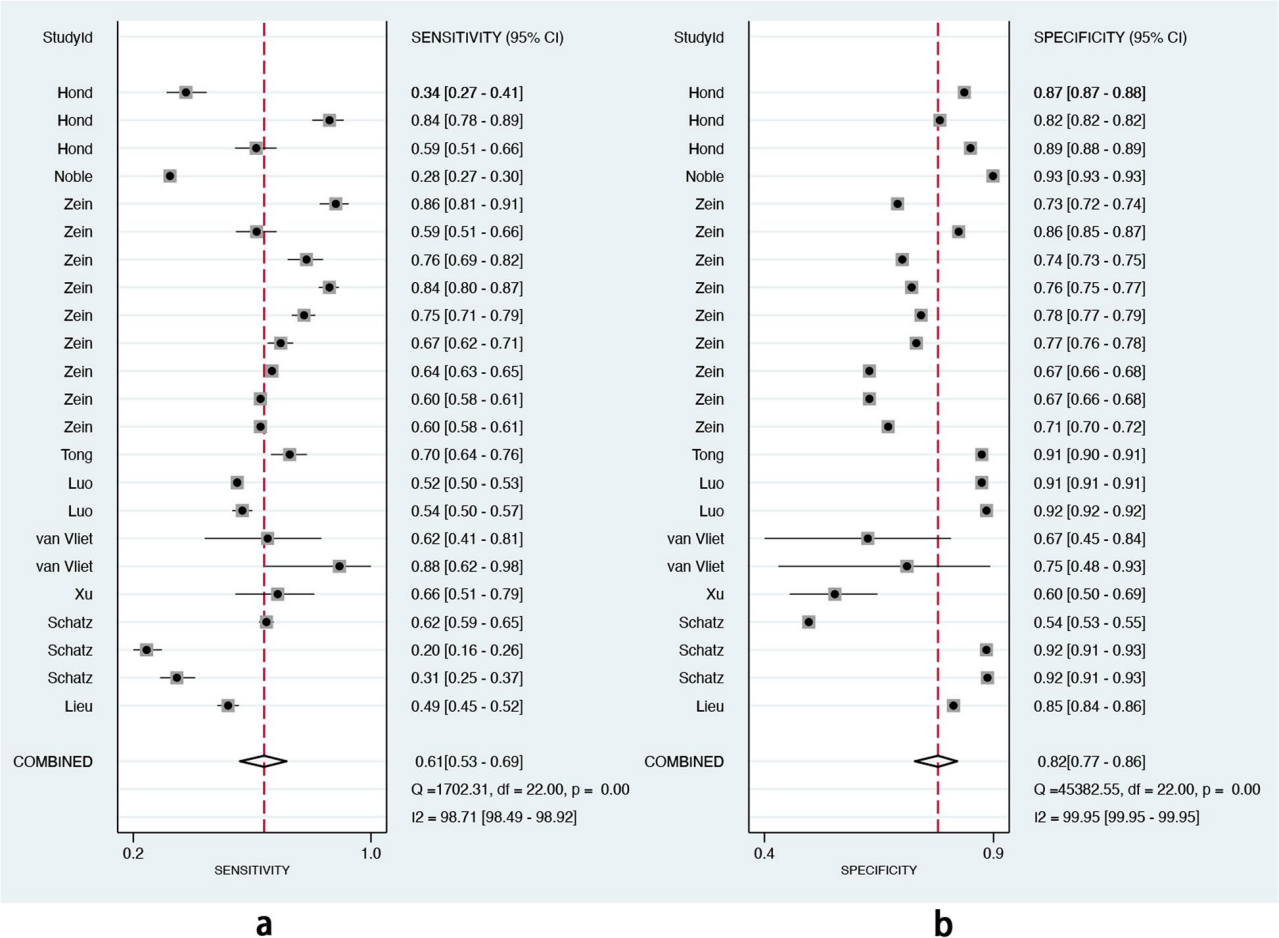
The overall pooled sensitivity (**a**) and specificity (**b**) of machine learning prediction models

We included 11 studies (23 models) with sufficient data and pooled performance measures of these studies in a random effects meta-analysis (see Additional file 7). The pooled AUROC for predicting asthma exacerbations was 0.80 (95% CI 0.76–0.83), indicating a good discrimination ability (Fig. 3). The pooled sensitivity and specificity were 0.61 (95% CI 0.53–0.69, *I*^2^ = 98.71, *P* < 0.01) and 0.82 (95% CI 0.77–0.86, *I*^2^ = 99.95, *P* < 0.01), respectively (Fig. 4). Other values were as follows: PLR 3.33 (95% CI 2.73–4.07, *I*^2^ = 99.58, *P* < 0.01), NLR 0.47 (95% CI 0.39–0.57, *I*^2^ = 98.89, *P* < 0.01), and DOR 7.02 (95% CI 5.20–9.47, *I*^2^ = 100.00, *P* < 0.01) (see Additional file 8).

**Fig. 5:**
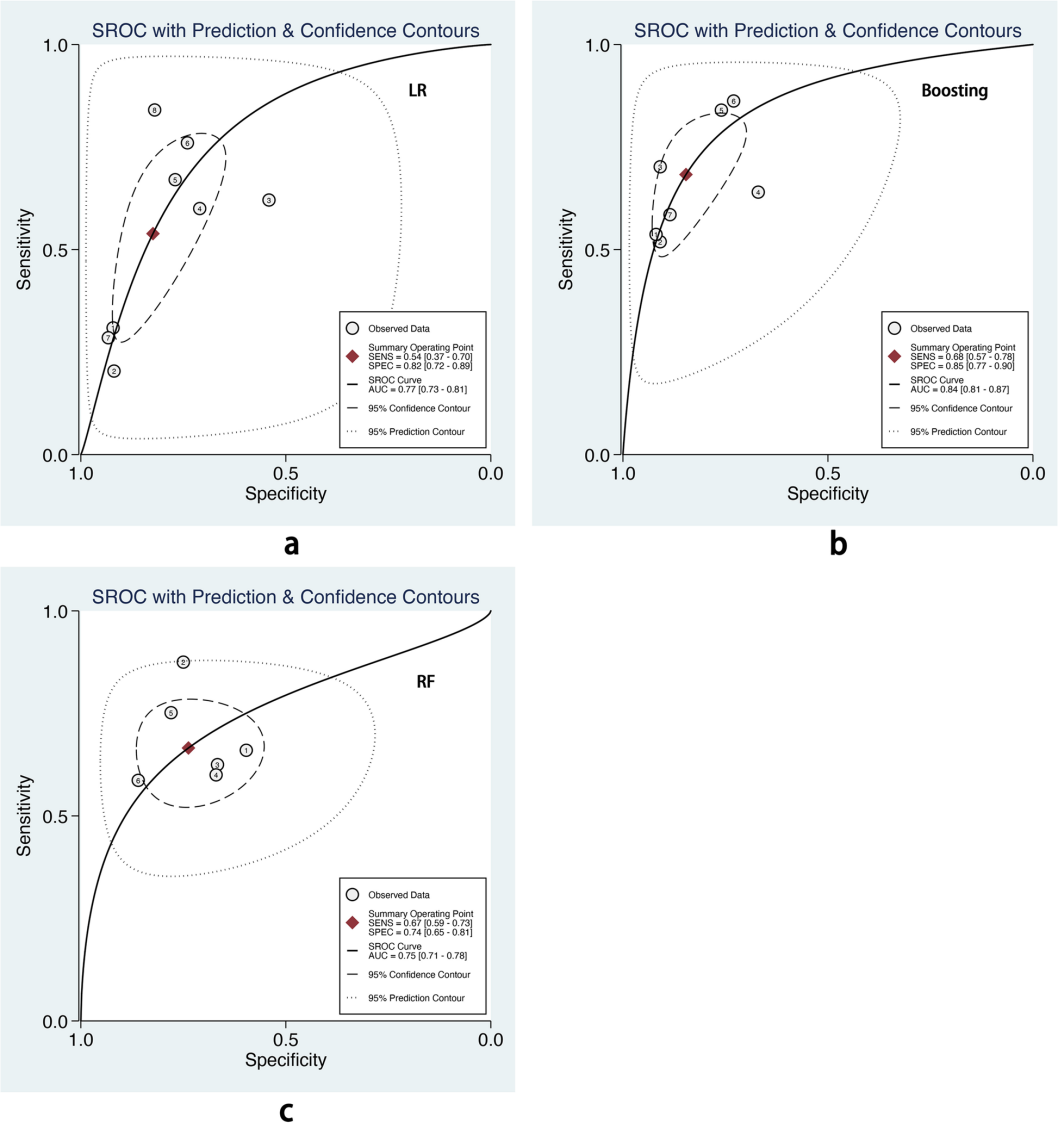
The overall pooled AUROC of machine learning prediction models stratified by logistic regression (**a**), boosting (**b**), and random forest (**c**) methods

**Fig. 6:**
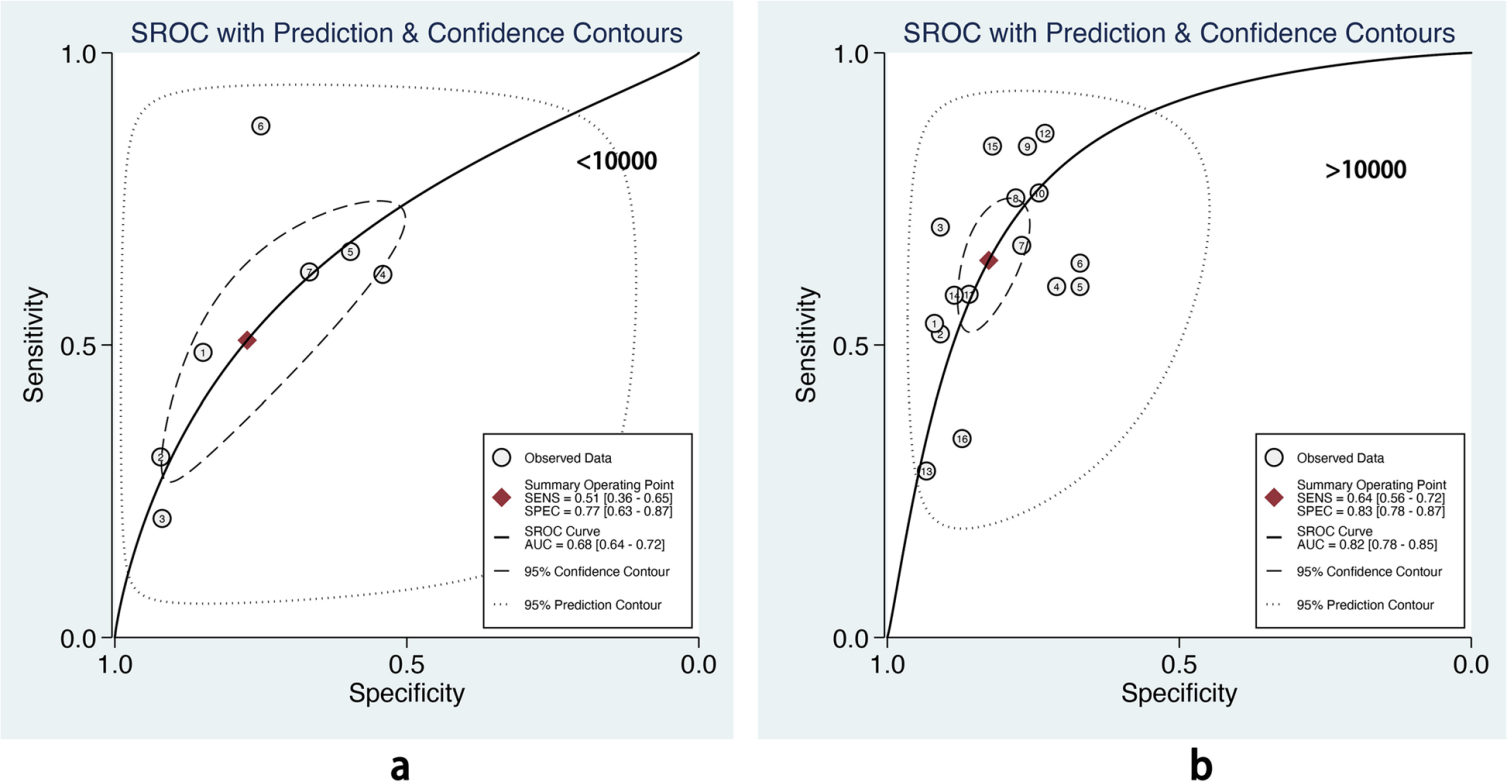
The overall pooled AUROC of machine learning prediction models stratified by different sample sizes. **a** Sample size < 10000. **b** Sample size > 10000

**Fig. 7:**
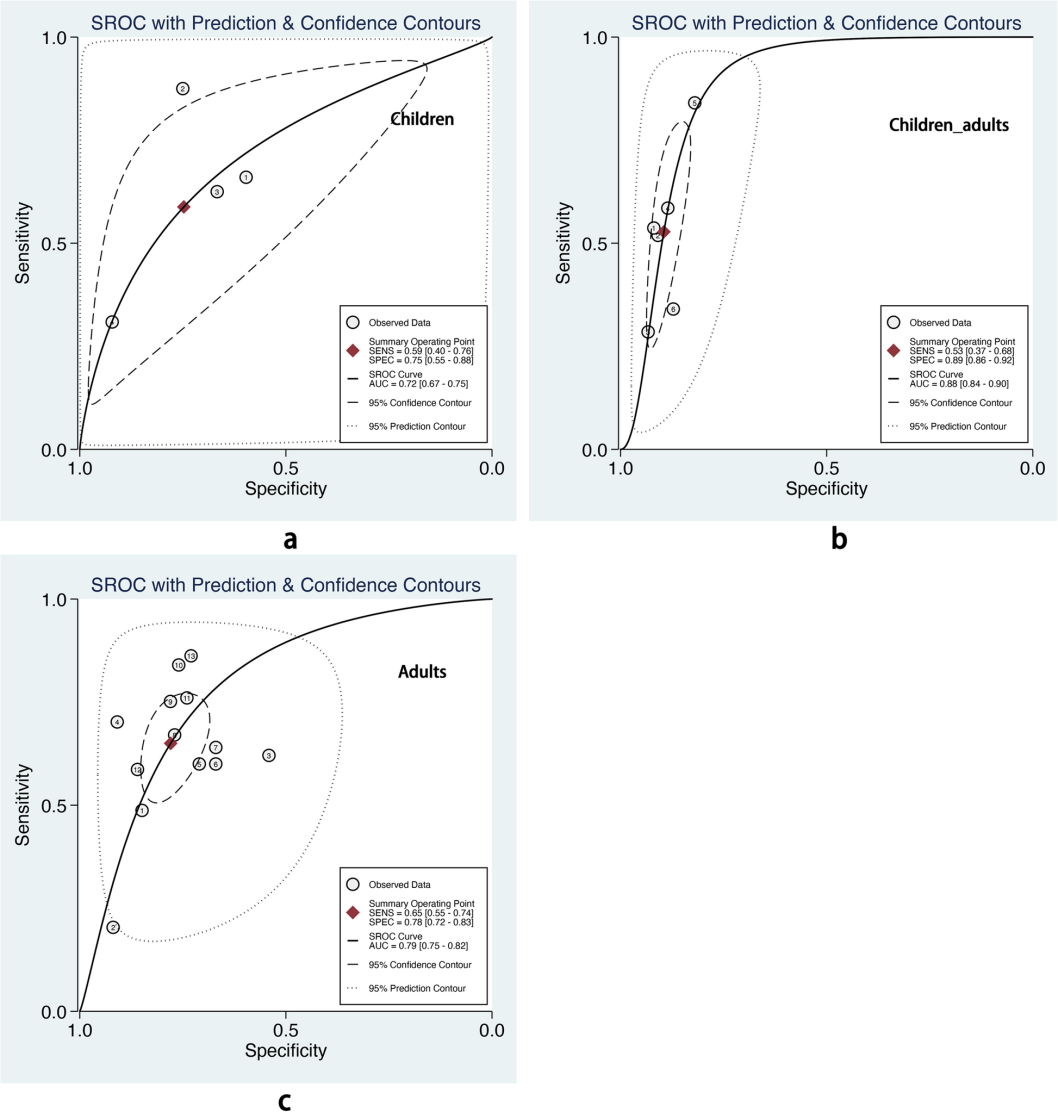
The overall pooled AUROC of machine learning prediction models stratified by different age groups. **a** Children. **b** Children and adults. **c** Adults

**Fig. 8:**
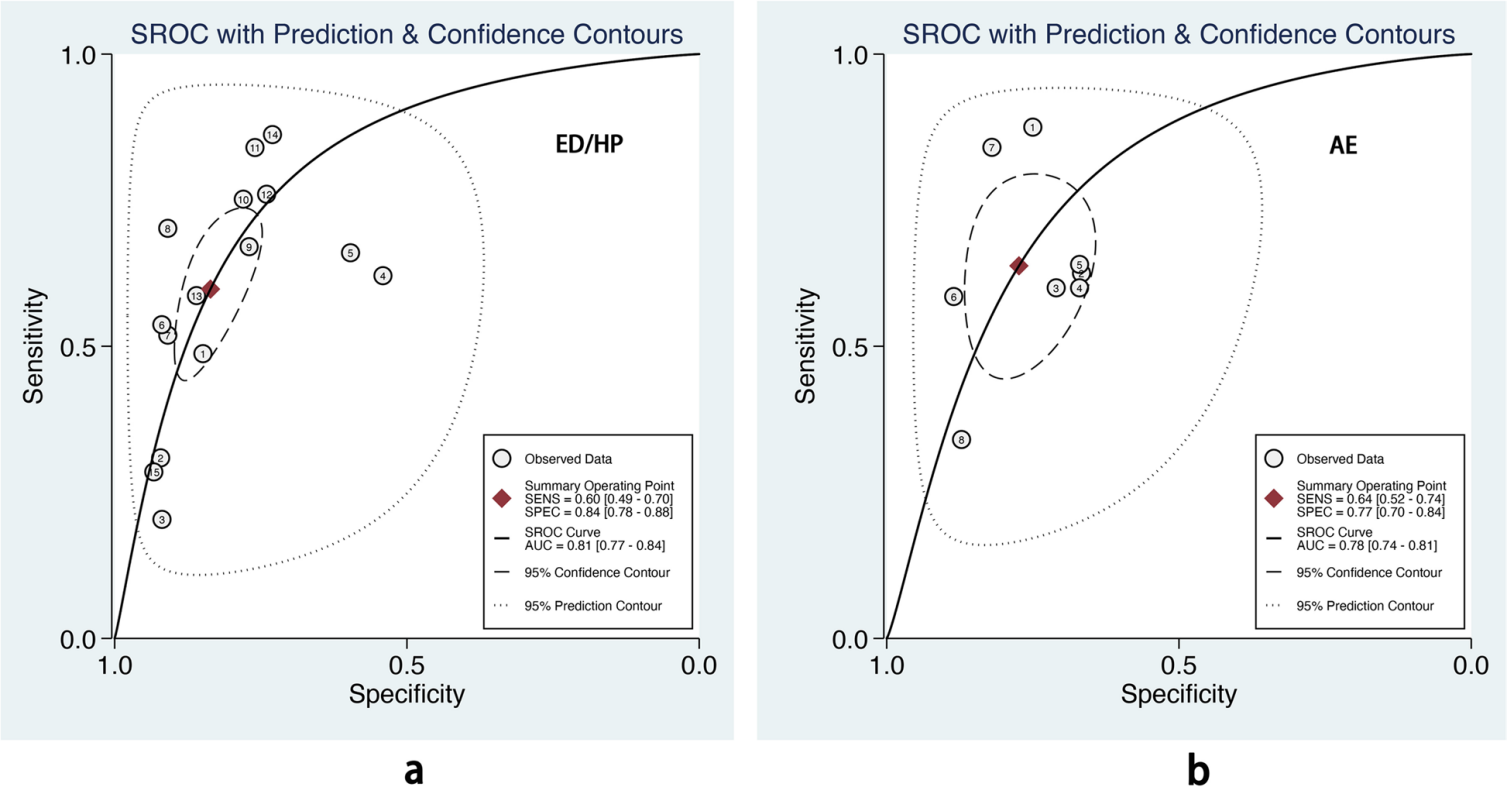
The overall pooled AUROC of machine learning prediction models stratified by different outcome events. **a** Emergency department visits/ hospitalization for asthma. **b** Asthma exacerbation definition in accordance with ATS/ERS statement

We also performed subgroup analysis stratified by ML methods, sample size, age group, and outcome definitions. As shown in Table 2, the overall pooled AUROC of boosting-based prediction models (0.84) was the highest, followed by studies using LR (0.77) and RF (0.75) (Table 2, Fig. 5). DOR, another measure of overall diagnostic ability, was also highest in studies applying boosting method (11.86). In studies with a large sample size (> 10000), the pooled AUROC and DOR were relatively high, with the number of 0.82 and 8.62, respectively (Table 2, Fig. 6). We classified outcome events as either emergency department visit/hospitalization (ED/HP) or in accordance with ATS/ERS statement (AE) definitions and performed subgroup analysis. The pooled AUROC in the two groups were similar, and the diagnostic odds ratio (DOR) was 7.58 for the ED/HP group and 6.01 for the AE group (Table 2, Fig. 7). Studies involving participants with children and adults had the highest pooled AUROC (0.88) and DOR (9.49) (Table 2, Fig. 8). Forest plots were shown in Additional file 9.

**Table 2:** The comparison of pooled performance measures in subgroups

	AUROC	sensitivity	specificity	PLR	NLR	DOR
*Machine learning(n)*						
LR (8)	0.77 (0.73–0.81)	0.54 (0.37–0.70)	0.82 (0.72–0.89)	3.06 (2.19–4.28)	0.56 (0.41–0.76)	5.47 (3.35–8.95)
Boosting (7)	0.84 (0.81–0.87)	0.68 (0.57–0.78)	0.85 (0.77–0.90)	4.44 (3.15–6.27)	0.37 (0.28–0.50)	11.86 (7.80–18.01)
RF (6)	0.75 (0.71–0.78)	0.67 (0.59–0.73)	0.74 (0.65–0.81)	2.54 (1.82–3.54)	0.45 (0.36–0.57)	5.59 (3.29–9.49)
*Sample size (n)*						
< 10000 (7)	0.68 (0.64–0.72)	0.51 (0.36–0.65)	0.77 (0.63–0.87)	2.24 (1.61–3.11)	0.64 (0.53–0.77)	3.52 (2.44–5.08)
> 10000 (16)	0.82 (0.78–0.85)	0.64 (0.56–0.72)	0.83 (0.78–0.87)	3.71 (3.00–4.58)	0.43 (0.35–0.53)	8.62 (6.25–11.89)
*Age group(n)*						
Children (4)	0.72 (0.67–0.75)	0.59 (0.40–0.76)	0.75 (0.55–0.88)	2.33 (1.54–3.51)	0.55 (0.41–0.74)	4.23 (2.72–6.57)
Children_adults(6)	0.88 (0.84–0.90)	0.53 (0.37–0.68)	0.89 (0.86–0.92)	5.02 (4.05–6.22)	0.53 (0.38–0.73)	9.49 (5.83–15.44)
Adults (13)	0.79 (0.75–0.82)	0.65 (0.55–0.74)	0.78 (0.72–0.83)	2.95 (2.32–3.75)	0.45 (0.35–0.57)	6.56 (4.37–9.86)
*Outcome(n)*						
ED/HP (15)	0.81 (0.77–0.84)	0.60 (0.49–0.70)	0.84 (0.78–0.88)	3.65 (2.86–4.65)	0.48 (0.38–0.61)	7.58 (5.35–10.74)
AE (8)	0.78 (0.74–0.81)	0.64 (0.52–0.74)	0.77 (0.70–0.84)	2.82 (2.06–3.86)	0.47 (0.35–0.63)	6.01 (3.47–10.41)

We perform the meta-regression analysis of the logit transformation of DOR due to the high level of heterogeneity. Univariate meta-regression analysis indicated that sample size and publication year contributed to the prediction power. However, only the coefficient of outcome definitions reached statistical significance in the multivariate model (Table 3). We included the outcome variable in the meta-regression analysis. The adjusted R-squared improved from 18.72% to 39.61%, and the Tau2 decreased from 0.4198 to 0.3118, indicating that the outcome variable could explain 25.7% heterogeneity.

**Table 3:** Univariate and multivariate outcomes of the random effects meta-regression

Variables	Univariate analysis				Multivariate analysis			
	Coeff	SE	*P*	95%CI	Coeff	SE	*P*	95%CI
Boosting	1.01	0.53	0.073	(-0.10, 2.12)	0.69	0.51	0.196	(-0.40, 1.78)
LR	0.22	0.52	0.670	(-0.87, 1.33)	0.01	0.47	0.987	(-1.00, 1.02)
RF	0.27	0.56	0.636	(-0.94, 1.44)	0.06	0.51	0.908	(-1.03, 1.15)
Sample size	0.83	0.31	0.016	(0.17, 1.48)	-0.89	0.96	0.365	(-2.93, 1.14)
Outcome	0.26	0.34	0.443	(-0.43, 0.96)	0.79	0.34	0.036	(0.06, 1.52)
Age group	0.16	0.18	0.394	(-0.22, 0.53)	0.27	0.17	0.134	(-0.09, 0.63)
Publication year	-0.39	0.17	0.037	(-0.75, -0.02)	-0.89	0.50	0.099	(-1.97, 0.19)

### Publication bias and sensitivity analysis

**Fig. 9:**
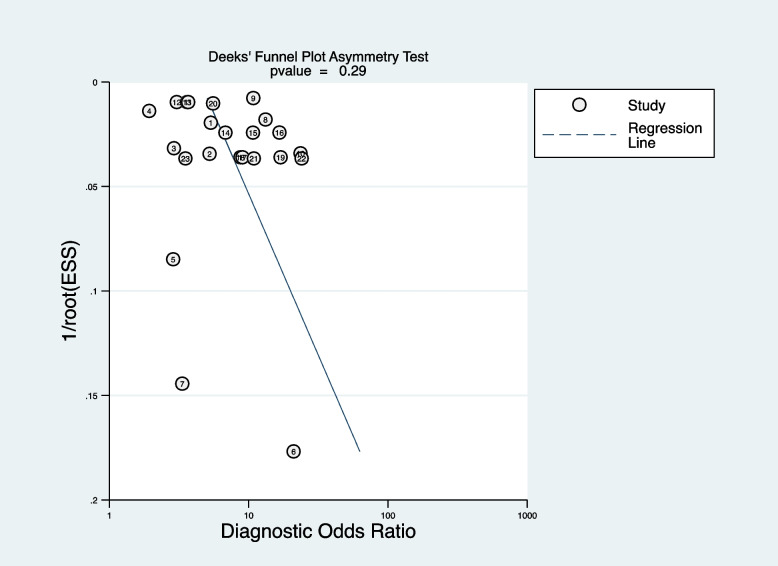
Deeks’ funnel plot of publication bias

Deeks’ funnel plot was applied to test publication bias. As shown in Fig. 9, the funnel plot was symmetrical, indicating no publication bias (*P* = 0.29). Sensitivity analysis showed exclusion of any study did not affect the pooled estimations, suggesting the stability of the meta-analysis (see Additional file 10).

## Discussion

### Principal findings

This study systematically reviewed the ML-based prediction models for asthma exacerbations, which have not been discussed before. Eleven studies generated 23 ML prediction models, which were various in study design, data source, participants, outcome definitions, and ML algorithms. 6/11 studies were published in the recent three years, indicating a popular trend in applying ML algorithms in asthma. The overall pooled AUROC (0.8, 95% CI 0.76–0.83) and DOR (7.02, 95% CI 5.20–9.47) indicated that ML-based prediction models for asthma exacerbation could achieve good discrimination. ML prediction models could forecast patients at high risk of exacerbation from several days to years, helping identify patients needing closer management.

LR, boosting, and RF are the top three popular algorithms for asthma exacerbation prediction. According to the subgroup analysis, boosting-based prediction models had the highest pooled AUROC with a pooled AUROC of 0.84(95% CI 0.81–0.87), and the confidence interval of pooled AUROC was non-overlap with LR (0.77, 95% CI 0.73–0.81) and RF (0.75, 95% CI 0.71–0.78). Bridge et al. [8] conducted a systemic review and found that LR had a higher pooled c-statistic than optimal action points and CART in predicting asthma exacerbations. However, the authors did not include other ML methods. In this article, we found that boosting could also achieve good performance. It was potentially an alternative method in asthma exacerbation prediction, and more candidate models developed by ML should be tested.

The sample size is crucial for model performance. Compared with robust techniques like LR and CART, modern ML methods need higher times of events per variable to achieve stable performance [25]. Our subgroup analysis also showed that compared with prediction models with a smaller sample size (< 10000 participants), models developed in a big sample size (> 10000 participants) showed relatively high pooled AUROC (0.82, 95% CI 0.78–0.85 vs. 0.68, 95% CI 0.64–0.72) in the test dataset. This suggests that ML methods would be preferable for prediction models only if a large dataset is available [25].

As for predictors, the most important features were systemic steroids, short-acting beta2-agonists, age, ED visit, asthma diagnosis number, exacerbation history, race, BMI, duration, blood eosinophils, and smoking. Most of these factors were consistent with the risk factor identified in GINA (https://ginasthma.org/wp-content/uploads/2021/04/GINA-2021-Main-Report_FINAL_21_04_28-WMS.pdf) and previous studies [26, 27]. Other biomarkers, such as volatile organic compounds and single nucleotide polymorphisms were also used as input features to predict asthma exacerbations [19, 20]. However, these studies were performed with a small sample size of participants resulting in a high risk of overfitting. In addition, these factors require advanced equipment, limiting application in practice. Socioeconomic factors were included in only three studies but were identified as insignificant. Environmental factors, such as air pollutants, are also crucial for asthma exacerbation [28]. However, none of these studies focus on environmental factors.

### Strengths and limitations

This study has several strengths. Firstly, we described included studies in detail and used logical methodology, which could provide a clear understanding of ML models in asthma exacerbation prediction. Additionally, the number of models allows us to conduct a meta-analysis of performance measures and compare different ML algorithms.

Despite the excellent prediction power of ML-based models confirmed in this study, several limitations are also identified. The main limitation was heterogeneity within studies. The difference in sample sizes, participants, feature selection, and prediction windows might affect the prediction ability of each model. Thus, the results analyzed in this study should be applied prudently. In addition, we did not include papers published in non-English, and we might not include all ML-based prediction models in the field of asthma exacerbations.

### Future direction

ML methods are a potential way to achieve excellent performance in asthma exacerbation prediction, and more ML methods should be tested in the future. Although many models were developed, few of them were applied in practice. Therefore, improving the generalizability of prediction models in large separate datasets is crucial. Practicability is another critical factor. Simple models with a few predictors and using predictors that are easy to access could improve prediction models' practicability. Moreover, bundling ML algorithms to software or system would benefit in translating research into practice applications. Besides, randomized control studies are warranted to evaluate whether these models could benefit asthmatic patients by preventing asthma exacerbations.

## Conclusion

Early identification of asthmatic patients at high risk of asthma exacerbations guides physicians to take closer management and timely intervention. This study showed that ML could achieve great performance in predicting asthma exacerbations. Future studies should focus on improving models' generalizability and practicability, thus driving the application of these models in clinical practice.

## Supplementary Information

**Additional file 1:** Search term and results.

**Additional file 2:** Methods.

**Additional file 3:** Definitions of participants and outcomes of included studies.

**Additional file 4:** The explanation of dataset split and validation methods.

**Additional file 5:** Features and most important features in prediction models.

**Additional file 6:** Risk of bias and applicability assessment based on PROBAST tools.

**Additional file 7:** 11 studies included in the meta-analysis.

**Additional file 8:** The overall pooled positive likelihood ratio, negative likelihood ratio, and diagnostic odds ratio of 23 machine learning prediction models.

**Additional file 9:** Forest plots of performance measures in subgroup analysis.

**Additional file 10:** The influence of each model for the outcome of meta-analysis

## Data Availability

All data generated or analyzed during this study are included in this published article and its supplementary information files.

## Abbreviations

ATS/ERS: Official American thoracic society/European respiratory society
CART: Classification and regression trees
DOR: Diagnostic odds ratio
GINA: Global initiative for asthma
LGBM: Light gradient boosting machine
LR: Logistic regression
ML: Mchine learning
NLR: Negative likelihood ratio
NN: Neural network
PLR: Positive likelihood ratio
PROBAST: The prediction model risk of bias assessment tool
RF: Random forest
ROB: Risk of bias
SVM: Support vector machine
