# Supplementary material for: Machine learning for prediction of asthma exacerbations among asthmatic patients: a systematic review and meta-analysis
Source: BMC Pulm Med. 2023 Jul 28;23:278. doi: 10.1186/s12890-023-02570-w (PMC10386701; doi:10.1186/s12890-023-02570-w)
Supplement: Supplementary file 1 — Additional file 1: Search term and results. [file 12890_2023_2570_MOESM1_ESM.pdf]

Additional file 1. Search term and results

PubMed<br>(-2022/12/15)<br>2103	#1	(((Asthma*[Title/Abstract]) OR (Bronchial Asthma[Title/Abstract])) OR (Asthma, Bronchial[Title/Abstract]))
	#2	(((((Acute*[Title/Abstract]) OR (Emergenc*[Title/Abstract])) OR (Exacerbation*[Title/Abstract])) OR (Attack*[Title/Abstract])) OR (Hospital*[Title/Abstract])) OR (Admission*[Title/Abstract])
	#3	(((((Status Asthmaticus[Title/Abstract]) OR (Asthmaticus, Status[Title/Abstract])) OR (Asthmatic Cris*[Title/Abstract])) OR (Cris*, Asthmatic[Title/Abstract])) OR (Asthmatic Shock*[Title/Abstract])) OR (Shock*, Asthmatic[Title/Abstract])
	#4	((((((((((((((((((((((((Machine learning[Title/Abstract]) OR (Deep learning[Title/Abstract])) OR (Support Vector Machine[Title/Abstract])) OR (SVM[Title/Abstract])) OR (Random forest[Title/Abstract])) OR (Logistic Model*[Title/Abstract])) OR (Logit Model*[Title/Abstract])) OR (Logistic Regression*[Title/Abstract])) OR (Adaboost[Title/Abstract])) OR (XGBoost[Title/Abstract])) OR (Catboost[Title/Abstract])) OR (Decision Trees[Title/Abstract])) OR (Naïve Bayes[Title/Abstract])) OR (Prediction model*[Title/Abstract])) OR (K nearest neighbor[Title/Abstract])) OR (KNN[Title/Abstract])) OR (Neural network*[Title/Abstract])) OR (Perceptron*[Title/Abstract])) OR (ANN[Title/Abstract])) OR (RNN[Title/Abstract])) OR (CNN[Title/Abstract])) OR (Long short-term memory[Title/Abstract])) OR (LSTM[Title/Abstract])) OR (FNN[Title/Abstract])) OR (Artificial intelligence[Title/Abstract])) OR (Natural language processing[Title/Abstract])) OR (NLP [Title/Abstract]))
	#5	#2 OR #3
	#6	#1 AND #4 AND #5
Cochrane Library<br>(- 2022/12/15)<br>193	#1	MeSH descriptor: [Asthma] explode all trees
	#2	(bronchial asthma): ti, ab, kw OR (Asthma, Bronchial): ti, ab, kw
	#3	#1 OR #2
	#4	(Acute*): ti, ab, kw OR (Emergenc*): ti, ab, kw OR (Exacerbation*): ti, ab, kw OR (Attack*): ti, ab, kw OR (Hospital*): ti, ab, kw
	#5	(Admission*): ti, ab, kw
	#6	#4 OR #5
	#7	MeSH descriptor: [Status Asthmaticus] explode all trees
	#8	(Asthmaticus, Status): ti, ab, kw OR (Asthmatic Cris*): ti, ab, kw OR (Cris*, Asthmatic): ti, ab, kw OR (Asthmatic Shock*): ti, ab, kw OR (Shock*, Asthmatic): ti, ab, kw
	#9	#7 OR #8
	#10	#6 OR #9
	#11	MeSH descriptor: [Machine learning] explode all trees
	#12	(Deep learning): ti, ab, kw OR (Support Vector Machine): ti, ab, kw

		OR (SVM): ti, ab, kw OR (Random forest): ti, ab, kw OR (Logistic Model*): ti, ab, kw
	#13	(Logit Model*): ti, ab, kw OR (Logistic Regression*): ti, ab, kw OR (*boost): ti, ab, kw OR (Decision Trees): ti, ab, kw OR (Naïve Bayes*): ti, ab, kw
	#14	(Prediction model*): ti, ab, kw OR (K nearest neighbor): ti, ab, kw OR (KNN): ti, ab, kw OR (Neural network*): ti, ab, kw OR (Perceptron*): ti, ab, kw
	#15	(ANN): ti, ab, kw OR (RNN): ti, ab, kw OR (CNN): ti, ab, kw OR (Long short-term memory): ti, ab, kw OR (LSTM): ti, ab, kw
	#16	(FNN): ti, ab, kw OR (Artificial intelligence): ti, ab, kw OR (Natural language processing): ti, ab, kw OR (NLP): ti, ab, kw
	#17	#11 OR #12 OR #13 OR #14 OR #15 OR #16
	#18	#3 AND #10 AND #17
Web of Science (-2022/12/15) 4085	#1	TS=(Asthma*) or TS=(Bronchial Asthma) or TS=(Asthma, Bronchial)
	#2	TS=(Acute*) or TS=(Emergenc*) or TS=(Exacerbation*) or TS=(Attack*) or TS=(Hospital*) or TS=(Admission*)
	#3	TS=(Status Asthmaticus) or TS=(Asthmaticus, Status) or TS=(Asthmatic Cris*) or TS=(Cris*, Asthmatic) or TS=(Asthmatic Shock*) or TS=(Shock*, Asthmatic)
	#4	#2 OR #3
	#5	TS=(Machine learning) OR TS=(Deep learning) OR TS=(Support Vector Machine) OR TS=(SVM) OR TS=(Random forest) OR TS=(Logistic Model*) OR TS=(Logit Model*) OR TS=(Logistic Regression*) OR TS=(*boost) OR TS=(Decision Trees) OR TS=(Naïve Bayes) OR TS=(Prediction model*) OR TS=(K nearest neighbor) OR TS=(KNN) OR TS=(Neural network*) OR TS=(Perceptron*) OR TS=(ANN) OR TS=(RNN) OR TS=(CNN) OR TS=(Long short-term memory) OR TS=(LSTM) OR TS=(FNN) OR TS=(Artificial intelligence) OR TS=(Natural language processing) OR TS=(NLP)
	#6	#1 AND #4 AND #5
EMBASE (-2022/12/15) 4083	#1	‘asthma’/exp
	#2	‘bronchial asthma’:ti,ab,kw OR 'asthma, bronchial':ti,ab,kw
	#3	#1 OR #2
	#4	‘acute*’:ti,ab,kw OR 'emergenc*':ti,ab,kw OR 'exacerbation*':ti,ab,kw OR ' attack*':ti,ab,kw OR 'hospital':ti,ab,kw OR 'admission*':ti,ab,kw
	#5	‘status asthmaticus’/exp
	#6	‘asthmaticus, status’:ti,ab,kw OR 'asthmatic cris*':ti,ab,kw OR 'cris*, asthmatic':ti,ab,kw OR 'asthmatic shock*':ti ab,kw OR 'shock*, asthmatic ':ti,ab,kw
	#7	#5 OR #6

	#8	#4 OR #7
	#9	‘machine learning’/exp
	#10	'deep learning':ti,ab,kw OR 'support vector machine':ti,ab,kw OR svm:ti,ab,kw OR 'random forest':ti,ab,kw OR 'logistic model*':ti,ab,kw OR 'logit model*':ti,ab,kw OR 'logistic regression*':ti,ab,kw OR adaboost:ti,ab,kw OR xgboost:ti,ab,kw OR catboost:ti,ab,kw OR 'decision trees':ti,ab,kw OR 'naïve bayes':ti,ab,kw OR 'prediction model*':ti,ab,kw OR 'k nearest neighbor':ti,ab,kw OR knn:ti,ab,kw OR 'neural network*':ti,ab,kw OR perceptron*:ti,ab,kw OR ann:ti,ab,kw OR rnn:ti,ab,kw OR cnn:ti,ab,kw OR 'long short-term memory':ti,ab,kw OR lstm:ti,ab,kw OR fnn:ti,ab,kw OR 'artificial intelligence':ti,ab,kw OR 'natural language processing':ti,ab,kw OR nlp:ti,ab,kw
	#11	#9 OR #10
	#12	#3 AND #8 AND #11
Total records 10434		
