# Supplementary material for: Machine learning for prediction of asthma exacerbations among asthmatic patients: a systematic review and meta-analysis
Source: BMC Pulm Med. 2023 Jul 28;23:278. doi: 10.1186/s12890-023-02570-w (PMC10386701; doi:10.1186/s12890-023-02570-w)
Supplement: Supplementary file 2 — Additional file 2: Methods. [file 12890_2023_2570_MOESM2_ESM.pdf]

Additional file 2. Methods

1. Elements of data extraction

Paper source	The first author's name, publication year
Study information	Country, study design (prospective, retrospective), data source, sample size, positive cases, negative cases
Prediction models	Machine learning algorithms, number of machine learning-based prediction models, validation methods, prediction windows, input features, most important features
Performance measures	Area under receiving operating curve (AUROC), specificity, sensitivity/recall/true positive rate, positive predictive value/precision, and negative predictive value)
Population characteristics	Age, gender, asthma status (if available)
Predictors	Demographic predictors, clinical predictors, social-economic predictors, environment predictors
Outcomes	Definitions

2. Commands for meta-analysis

```
# Pool performance measure
midas tp fp fn tn, res(all)
# SROC and pooled AUROC
midas tp fp fn tn, plot sroc(both)
#Meta-regeression analysis
metareg lndor variable_list, wsse(Selndor) bsest(reml)
#Forest plots of sensitivity and specificity
midas tp fp fn tn, id(author) table(dss) texts(0.60) bfor(dss) ford fors ms(0.75)
# Forest plots of positive and negative likelihood ratio
midas tp fp fn tn, id(author) table(dlr) texts(0.70) ford fors bfor(dss)
# Forest plots of diagnostic odds ratio
midas tp fp fn tn, id(author) table(dlor) ford fors bfor(dss) texts(0.70)
# Publication bias test
midas tp fp fn tn, pubbias
```
