# Supplementary material for: Machine learning for prediction of asthma exacerbations among asthmatic patients: a systematic review and meta-analysis
Source: BMC Pulm Med. 2023 Jul 28;23:278. doi: 10.1186/s12890-023-02570-w (PMC10386701; doi:10.1186/s12890-023-02570-w)
Supplement: Supplementary file 3 — Additional file 3: Definitions of participants and outcomes of included studies. [file 12890_2023_2570_MOESM3_ESM.pdf]

Additional file 3. Definitions of participants and outcomes of included studies

Studies (author, year)	Participants	Outcome
Lieu, 1999	**Asthma**<br>1. A hospitalization, ED visit, or outpatient clinic visit with an ICD-9 code of 493.XX<br>2. Adult aged 18-44 years who used asthma medications<br>3. Exclude adults aged 45 and older unless they had an ICD-9-coded diagnosis of asthma	**Asthma-related hospitalization or ED visit:** a principal diagnosis of 493.XX; 2. Any diagnosis of 493.XX + a principal diagnosis of an asthma-related respiratory condition; Asthma-related ED visit: a diagnosis of asthma or a related respiratory condition
Schatz, 2004	**Asthma**<br>1. An ICD-9 code of 493.XX<br>2. ≥ 2 asthma-related medication dispensing in 1 year<br>3.Any ED or regular clinic asthma-related visit in the diagnosis and procedures database<br>4. 5-56 years old	**Asthma hospitalization** (primary diagnosis of asthma, ICD code of 493.xx) **or ED visit for asthma.**
Schatz, 2006	**Asthma**<br>1. A hospital discharge principal diagnosis of asthma (ICD-9code 493.xx).<br>2. An ED visit with asthma as the principal diagnosis (ICD-9code 493.xx).<br>3. Four or more asthma-related medication dispensing	**Emergency hospital utilization** for asthma: at least 1 hospitalization or ED visit for asthma (ICD-9 code of 493.xx).
Xu, 2011	**Asthma (mild-moderate):**<br>1. Asthma symptoms and/or medication use for ≥ 6 months in the previous year<br>2. Airway responsiveness with a provocative concentration	**Severe asthma exacerbation:** an emergency room visit or a hospitalization for asthma symptoms (questionnaire during screening visits).

	dose (PC20) of methacholine ≤ 12.5mg/ml.	
van Vliet, 2017	**Asthma:**<br>1. 6-18yr<br>2. Diagnosed based on GINA and the guidelines of the Dutch Society of Pediatrics	**Moderate to severe asthma exacerbation:** based on the criteria of the ATS/ERS recommendation.
Luo, 2020	**Asthma:**<br>Patient had one or more diagnosis codes of asthma (ICD-10 code of J45.x; ICD-9 code of 493.0x, 493.1x, 493.8x,493.9x).	**An asthma-related hospital encounter**: ED visit or hospitalization with asthma as the principal diagnosis (ICD-10: J45.x; ICD-9: 493.0x,493.1x, 493.8x, 493.9x).
Luo, 2020	**Asthma:**<br>Patient had one or more diagnosis codes of asthma (ICD-10 code of J45.x; ICD-9 code of 493.0x, 493.1x, 493.8x,493.9x).	**An asthma-related hospital encounter**: ED visit or hospitalization with asthma as the principal diagnosis (ICD-10: J45.x; ICD-9: 493.0x,493.1x, 493.8x, 493.9x).
Tong, 2021	**Asthma**<br>1. ≥ 18yr<br>2. ≥ 1 asthma diagnosis code (ICD-9 code of 493.0x, 493.1x, 493.8x, 493.9x; ICD-10 code of J45.x)	**Asthma hospital encounter:** a hospitalization or an ED visit that has asthma as its principal diagnosis (ICD-9: 493.0x, 493.1x, 493.8x, 493.9x; ICD-10: J45.x)
Zein, 2021	**Asthma:**<br>1.18-80 yr<br>2. ICD9 codes of 493.xx or TenthRevision of J45.xx	**Nonsevere asthma exacerbation:** oral glucocorticoid burst<br>**Severe asthma exacerbations:** Requiring an ED visit or hospitalization for asthma
Noble, 2021	**Asthma:**<br>1. 12-80 yr<br>2. Physician-diagnosed and recorded asthma	**≥1 hospitalization(s) within 12 months**
Hond, 2022	**Asthma (stable mild-moderate):**<br>1.12-75 yr<br>2. Stable asthma: exclude patients with ≥ 4 courses of oral prednisone in the previous 12 months, admission to the hospital because of asthma in the previous 6 months or to the	**Severe asthma exacerbations**: the need for a course of oral corticosteroids (prednisone) for a minimum of 3 days, as documented in medical records

	intensive care unit because of asthma at any time in the past	

ATS/ERS: American Thoracic Society and European Respiratory Society
ICD: International Classification of Diseases
GINA: Global Initiative for Asthma
