# Supplementary material for: Machine learning for prediction of asthma exacerbations among asthmatic patients: a systematic review and meta-analysis
Source: BMC Pulm Med. 2023 Jul 28;23:278. doi: 10.1186/s12890-023-02570-w (PMC10386701; doi:10.1186/s12890-023-02570-w)
Supplement: Supplementary file 4 — Additional file 4: The explanation of dataset split and validation methods. [file 12890_2023_2570_MOESM4_ESM.pdf]

Additional file 4. The explanation of dataset split and validation methods

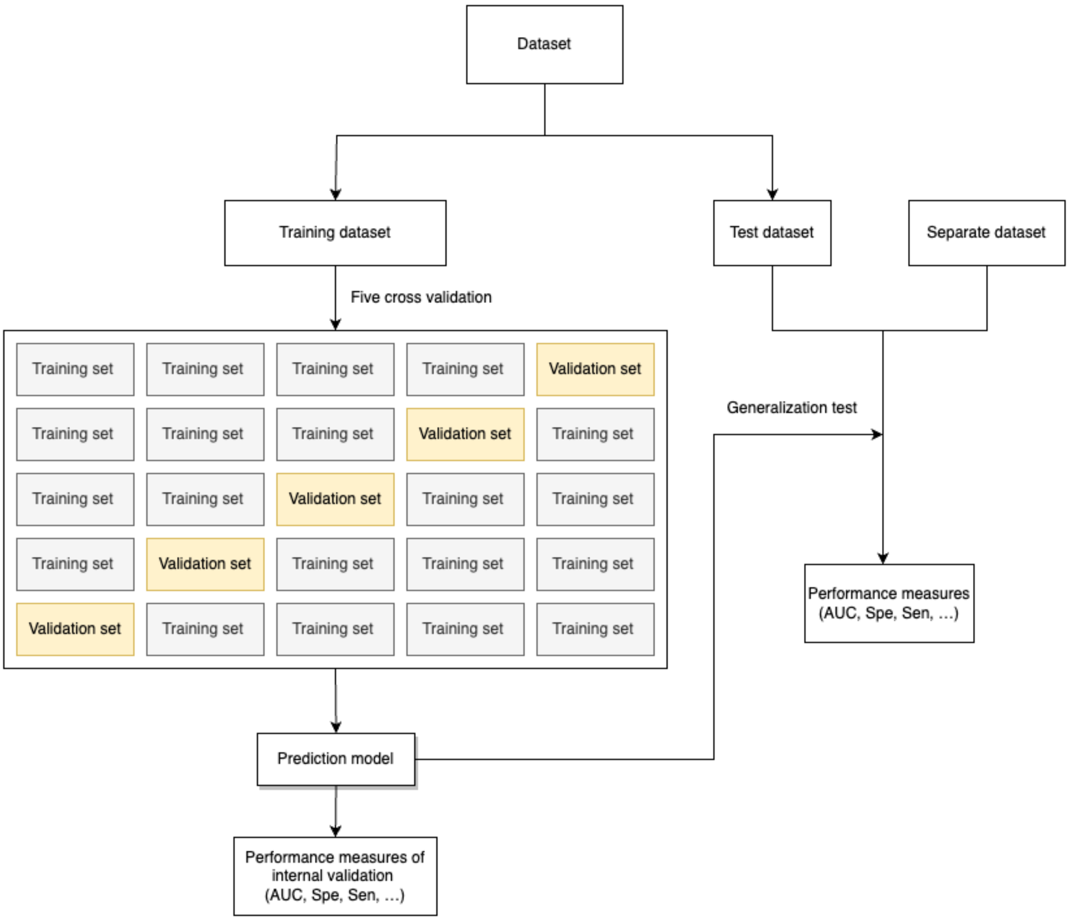

Training data is used to train machine learning algorithms. Models create and refine their rules using this data. It is a set of data samples used to fit the parameters of a machine learning model to train it by example.
Internal validation: Performance measures calculated from a dataset split from the training dataset called internal validation, such as cross-validation.
External validation: Performance test in a separate dataset called external validation, such as temporal validation (these data may be collected by the same investigators, commonly using the same predictor and outcome definitions and measurements but sampled from a later period) and geographic validation (these data may be collected by other investigators in another hospital or country, sometimes using different definitions and measurements).
Randomly splitting a single data set into a development and a validation data set (test dataset in the above figure) is often erroneously referred to as a form of external validation. Actually, it is an inefficient form of internal validation.
Generalization is the process of learning from data and making predictions about new, unseen data. A machine learning model is said to generalize well if it can make accurate predictions on new data, even data that is different from the training data.

Reference
1. Moons KGM, Wolff RF, Riley RD, Whiting PF, Westwood M, Collins GS, et al. PROBAST: A tool to assess risk of bias and applicability of prediction model studies: Explanation and elaboration. Annals of Internal Medicine. 2019;170: W1–33.
