# Supplementary material for: Machine learning for prediction of asthma exacerbations among asthmatic patients: a systematic review and meta-analysis
Source: BMC Pulm Med. 2023 Jul 28;23:278. doi: 10.1186/s12890-023-02570-w (PMC10386701; doi:10.1186/s12890-023-02570-w)
Supplement: Supplementary file 5 — Additional file 5: Features and most important features in prediction models. [file 12890_2023_2570_MOESM5_ESM.pdf]

Additional file 5. Features and most important features in prediction models

Studies (author, year)	Features used in final model				Top 10 important features
	Demographic	Clinical	Social-economic	Other factors	
Lieu, 1999	√	√			Oral steroid prescription (past 12m); ED visit (past 6m); Asthma medication prescription (past 6m); anti-inflammatory/beta2-agnoist (past 6m)
Schatz, 2004		√			ED visit or hospitalization; any oral corticosteroids; >14 beta agonist canisters
Schatz, 2006		√			Medication intensity Scale (include two elements: beta agonist canisters and oral corticosteroid)
Xu, 2011	√	√			SNPs
van Vliet, 2017		√			7 volatile organic compounds
Luo, 2020	√	√	√	Facility feature; primary care provider features	Asthma visit number; systemic steroid ordered; the number of days since the last ED visit; age; the last visit's admission type; asthma duration; ED visit number; SABA ordered
Luo, 2020	√	√	√	Primary care provider features	Nebulizer medications filled; asthma reliever orders; days since last asthma exacerbation or status asthmaticus diagnosis; ED visit for asthma; ED visits; age; primary/principal asthma diagnoses
Tong, 2021	√	√	√	Primary care provider feature	Asthma related ED visit number; length of stay of an ED visit; days since the most recent ED visit; primary/principal asthma diagnoses; days since last asthma exacerbation or status

					asthmaticus diagnosis; race; ED visit number; asthma diagnosis number; asthma duration; nebulizer medication orders
Zein, 2021	√	√			Non-severe: ICS+LABA; sinusitis; LTRA; nasal steroids; BMI; eosinophil; albumin; age; IgE; gastroesophageal reflux disease
					ED: oral steroid burst; age; race; ICS+LABA; albumin; eosinophil; BMI; creatinine; Hispanic; smoking status
					HP: oral steroid burst; creatinine; ICS+LABA; haemoglobin; BMI; LTRA; eosinophil; race; hypertension; albumin
Noble, 2021	√	√			BMI; smoking status; blood eosinophil counts; diabetes diagnosis/therapy; ischemic heart disease diagnosis; anaphylaxis history; GINA management step; consultation for LRTIs; acute oral steroids; hospitalization number
Hond, 2022		√			PEF; nocturnal awakening; beta2 reliever

Demographic factors: age, sex, and race.
Clinical factors: medical history, symptoms, comorbidities, signs, auxiliary examination, medicine, visit types/status, et al.
Social economic factors: insurance, income, population information, residence, et al
ICS: Inhaled corticosteroid; LABA: Long-acting β2-agonists; LTRA: Leukotriene-receptor antagonists; SAAC: Short-acting anticholinergics; SABA: Short-acting β2-agonists; LRTI: Lower respiratory tract infection; PEF: peak expiratory flow.
