# Supplementary material for: Machine learning for prediction of asthma exacerbations among asthmatic patients: a systematic review and meta-analysis
Source: BMC Pulm Med. 2023 Jul 28;23:278. doi: 10.1186/s12890-023-02570-w (PMC10386701; doi:10.1186/s12890-023-02570-w)
Supplement: Supplementary file 6 — Additional file 6: Risk of bias and applicability assessment based on PROBAST tools. [file 12890_2023_2570_MOESM6_ESM.pdf]

Additional file 6. Risk of bias and applicability assessment based on PROBAST tools.

Studies	Risk of bias				Applicability			Overall	
	Participants	Predictors	Outcome	Analysis	Participants	Predictors	Outcome	ROB	Applicability
Lieu, 1999	High	Low	High	High	Low	Low	High	High	High
Schatz, 2004	High	Low	Low	High	Low	Low	High	High	High
Schatz, 2006	High	Low	Low	High	Low	Low	High	High	High
Xu, 2011	Low	Low	High	High	Low	High	High	High	High
van Vliet, 2017	Low	Low	Low	High	Low	Low	Low	High	Low
Luo, 2020	High	High	Low	High	Low	High	High	High	High
Luo, 2020	High	High	Low	High	Low	High	High	High	High
Tong, 2021	High	High	Low	High	Low	High	High	High	High
Zein, 2021	High	High	High	High	Low	High	High	High	High
Noble, 2021	High	High	High	High	Low	High	High	High	High
Hond, 2022	Low	Low	Low	High	Low	Low	High	High	High
