# Supplementary material for: Machine learning for prediction of asthma exacerbations among asthmatic patients: a systematic review and meta-analysis
Source: BMC Pulm Med. 2023 Jul 28;23:278. doi: 10.1186/s12890-023-02570-w (PMC10386701; doi:10.1186/s12890-023-02570-w)
Supplement: Supplementary file 7 — Additional file 7: 11 studies included in the meta-analysis. [file 12890_2023_2570_MOESM7_ESM.pdf]

Additional file 7. 11 studies included in the meta-analysis.

Study	Study design	Sample size(train)	Sample size(test)	Outcome	ML methods	Features in final model	TP	TN	FP	FN
Lieu 1999	Retrospective	7141	10000	HP/ED for asthma	CART	Oral steroid prescription (past 12m), ED visit (past 6m), Asthma medication prescription (past 6m), anti-inflammatory/beta2-agnoist (past 6m)	346	7887	1403	364
Schatz 2004	Retrospective	8789	6104	HP/ED for asthma	LR	ED visit or hospitalization, any oral corticosteroids, >14 beta agonist canisters	122	5172	450	360
			2585(children)				72	2167	185	161
			3519(adults)				55	2986	263	215
Schatz 2006	Retrospective	1079	24370	HP/ED for asthma	LR	Medication intensity Scale (include two elements: beta agonist canisters and oral corticosteroid)	861	12380	10503	526
Xu 2011	Retrospective	417	164	Severe AE	RF	160 SNPs, age, male, FEV1%, treatment	33	68	46	17
van Vliet 2017	Prospective	574	32	AE in 14 days	RF	7 volatile organic compounds	14	12	4	2
	Prospective	574	48	AE in 20 days	RF		15	16	8	9
Luo 2020	Retrospective	315308	19256	HP/ED for asthma	XGBoost	142 features*	436	16955	1489	376
Luo 2020	Retrospective	782762	204744	HP/ED for asthma	XGBoost	221 features†	2259	182176	18215	2094
Tong 2021	Retrospective	68244	14644	HP/ED for asthma	XGBoost	71 features§	153	13115	1311	65
Zein 2021	Retrospective	48242	12060	Nonsevere asthma	LR	Age, gender, BMI, Race, Hispanic, Alcohol abuse, Anemia, Coagulopathy, Depression, Drug Abuse, GERD, Liver Disease, Menopause, Nasal Polyps, Peptic Ulcer, Psychoses, Rheumatoid arthritis collagen vascular diseases Sleep Apnea, UC, Albumin, Creatinine Eosinophil FeNO IgE, Testosterone, Oral Glucocorticoids Bursts (frequency), Chronic Steroid, iCS +, LABA or HDiCS, Nasal Steroids, Theophylline, Sinusitis, Malignancy without metastasis, Smoking Status, Hypothyroidism, AIDS, Bilirubin, Blood_Loss, DHEAS, CROHNS_DISEASE, Estradiol, Diabetes, Hgb, Intubation, Progesterone, Hypertension, Nasal	2487	5620	2295	1658
	Retrospective	48242	12060	Nonsevere asthma	RF		2487	5303	2612	1658
	Retrospective	48242	12060	Nonsevere asthma	LGBM		2653	5303	2612	1492
	Retrospective	48242	12060	ED for asthma	LR		297	8944	2673	146
	Retrospective	48242	12060	ED for asthma	RF		333	9060	2556	110
	Retrospective	48242	12060	ED for asthma	LGBM		373	8828	2788	71
	Retrospective	48242	12060	HP for	LR		149	8780	3084	47

				asthma		Antihistamines, Lymphoma, Anit-IgE, Metastatic cancer, iCS, Paralysis, LTRAs, Peripheral vascular disease, SABA, Pulmonary Circulation Disorders, Renal Failure				
	Retrospective	48242	12060	HP for asthma	RF		115	10203	1661	81
	Retrospective	60302	12060	HP for asthma	LGBM		169	8661	3203	27
Noble 2021	Retrospective	58619	174240	HP for asthma	LR	Age, BMI, smoking status, blood eosinophils counts, rhinitis, diabetes, IHD diagnosis, anxiety/depression, anaphylaxis history, GINA step, GP consultation for LRTIs, acute oral steroids, paracetamol, hospitalization	695	160290	11511	1744
Hond 2022	Retrospective	92787	40092	Severe asthma	XGBoost	Nocturnal awakening, the average of morning and evening PEF, the use of β2-reliever in morning and evening, PEF average, standard deviation, maximum and minimum, PEF personal best, first differences and lags for PEF, nocturnal awakening, and use of β2-reliever	110	35326	4578	78
	Retrospective	92787	40092	Severe asthma	LR		158	32720	7184	30
	Retrospective	92787	40092	Severe asthma	One class SVM		64	34751	5153	124

*: The number of major visits for asthma, The total number of units of systemic corticosteroids ordered, The number of days since the last ED visit, Age, The last visits admission type = elective, Duration of asthma, The number of ED visits, The total number of units of short-acting beta-2 agonists ordered, The total number of short-acting beta-2 agonists ordered, The total number of systemic corticosteroids ordered, The maximum blood eosinophil count, The maximum percentage of blood eosinophils, The number of ICD-9 and ICD-10 procedure codes, The number of distinct asthma medication prescribers, The average respiratory rate, The average heart rate, The total number of units ordered in all of the medication orders, The proportion who incurred hospital encounters for asthma in the index year out of all asthmatic patients of the PCP in the year before, Ethnicity, Whether nebulizer was used, The time between making the request and the actual visit of the last visit, The number of asthma medication orders, The number of ICD-9 and ICD-10 diagnosis codes, The total number of distinct medications in all of the medication orders, The total number of units of asthma medications ordered, The block groups national health literacy score, The total number of distinct medications in all of the asthma medication orders, The block groups median family income , Marital status = married, The number of outpatient visits, The percentage of families below 150% of the federal poverty level in the block group, The total number of medications in all of the medication orders, The maximum BMI, The shortest time between making the request and the actual visit among all of the visits, The number of laboratory tests with abnormal results, The number of distinct providers seen in outpatient visits, The average diastolic blood pressure, The ellipsoid great circle distance between the patients home and the closest ED, The change of BMI in percentage, The total number of units of inhaled corticosteroids ordered, Singhs area deprivation index of the block group, The number of insurances of the patient at the last visit, The areas black population percentage, Race = white, The average length of an inpatient stay, The percentage of population 25 and older with a high school diploma or higher education in the block group, The block groups income disparity measure, The areas white population percentage, The percentage of employed people 16 and older in the block group who are in a white-collar occupation, The areas average house value, The number of allergies of the patient, The number of families in the block group, The number of distinct medication prescribers, The change of weight in percentage, The ellipsoid great circle distance between the patients home and the patients current PCPs office, The average SpO2, The areas Hispanic population percentage, Race = Asian, The number of days since the last outpatient visit, The total number of refills allowed in all of the medication orders, The block groups median monthly rent payment, The number of laboratory tests, The admit hour of the last ED visit, Gender, The combined population of each of the census blocks within the block group that qualifies as urban under the 2013 US census, The percentage of single-parent households with dependents <18 years old in the block group, Bronchiolitis, The maximum temperature, The block groups median monthly mortgage payment, The total length of all of the inpatient stays, The number of asthmatic patients of the PCP, Religion = Protestant, The percentage of the civilian labor force 16 and older in the block group that is unemployed, The percentage of households in the block group that are owner-occupied, The number of civilian labor force 16 and older in the block group, Whether the patient had any food allergy, The PCPs age, The minimum SpO2, The estimated average number of people per household in the area, Religion = Catholic, The number of households in the block group, The average systolic blood pressure, The average temperature, The areas population size, The total number of units of asthma reliever medications ordered that are neither systemic corticosteroids nor short-acting beta-2 agonists, The number of chest X-ray exams, The percentage of households in the block group without a motor vehicle, The number of medication orders, The areas average household income, The size of the population 25 and older in the block group , The areas average elevation, The percentage of households in the block group with >1 person per room, The primary payers insurance category at the patients last visit = other private insurance, Smoking status = current smoker, The number of no shows, Whether the last inpatient stay was through the ED, Whether the patient had any drug or material allergy, The number of CPT procedure codes for pulmonary function tests, The maximum respiratory rate, The number of CPT/HCPCS procedure codes, The acuity level of the last ED visit, The number of days since having the last laboratory test, The median home value in the block group, The number of occupied households in the block group, Religion = Christian, The maximum heart rate, The primary payers insurance category at the patients last visit = SelectHealth, The percentage of population 25 and older in the block group with < 9 years of education, The PCPs primary specialty = family medicine, The number of employed people 16 and older in the block group, The areas 2003 rural-urban continuum code, The percentage of families in the block group that are below the federal poverty level, The percentage of households in the block group without a phone, The length of stay of the last ED visit, Diabetes without chronic complication, The number of days since the last inpatient stay, The day of the week at the last ED visits admission time, The maximum diastolic blood pressure, The total number of refills allowed in all of the short-acting beta-2 agonist orders, Religion = Baptist, Smoking status = former smoker, The number of cancelled appointments, The estimated number of households in the area, The PCPs primary profession type = Doctor of Osteopathic Medicine, Whether the patient had any environmental allergy, The total number of refills allowed in all of the inhaled corticosteroid orders, The maximum systolic blood pressure, The total number of units of NSAIDs ordered, Among the admission types of

all of the visits of the patient, the one with the highest priority = urgent, Chronic obstructive pulmonary disease, Language = Spanish, Obesity, Marital status = single, Upper respiratory tract infection, The number of outpatient visits to the patients PCP, The length of the last intensive care unit stay, The number of visits that were referred, The total number of refills allowed in all of the nasal steroid spray orders, Breathing abnormality like dyspnea, The block groups rural/urban status, The total number of antihistamines ordered, The duration of chronic obstructive pulmonary disease

†: Total # of units of nebulizer medications filled, # of asthma reliever orders, Total # of asthma relievers ordered, # of days since having the last diagnosis of asthma with (acute) exacerbation or status asthmaticus, # of ED visits on asthma, # of nebulizer medication orders, # of ED visits, # of ED visits on asthma in the pre-index year, Age, # of primary or principal asthma diagnoses, # of ED visits in the pre-index year, Total # of asthma relievers filled, Total # of units of nebulizer medications filled in the pre-index year, # of major visits for asthma in the pre-index year, The highest exacerbation severity of all of the asthma diagnoses in the pre-index year, # of nebulizer medication orders in the pre-index year, # of days since the last ED visit on asthma, # of ED visits on asthma in the pre-pre-index year, The day of the week of the last ED visits admission time, The highest exacerbation severity of all of the asthma diagnoses, Total # of short-acting beta-2 agonists ordered, # of ED visits in the pre-pre-index year, Whether the patient is black or African American, Total copay for medications in the pre-pre-index year, # of primary or principal asthma diagnoses in the pre-index year, # of asthma reliever orders in the pre-index year, # of active problems of asthma, # of asthma diagnoses, # of major visits for asthma, # of days since the last use of asthma relievers, Total copay for medications, The highest exacerbation severity of all of the asthma diagnoses in the pre-pre-index year, # of primary or principal asthma diagnoses in the pre-pre-index year, Total # of units of nebulizer medications filled in the pre-pre-index year, Total # of units of asthma relievers filled in the pre-index year, Total # of units of medications filled, # of virtual visits by email, Whether the patient had access to kp.org, # of no shows, # of active problems of asthma with (acute) exacerbations, The average respiratory rate, # of visits with same day appointments, The average heart rate, The maximum temperature, # of asthma medication orders in the pre-index year, The average SpO2, Whether the last visits admission type is elective, # of asthma reliever orders in the pre-pre-index year, # of nebulizer medication orders in the pre-pre-index year, Whether the patient is divorced, The areas median household income, Total copay for medications in the pre-index year, # of days since having the last asthma diagnosis, The areas percentage of household income that is between 150,000 and $199,999, Whether the most emergent admission type of all of the visits is emergency, # of years for which the patient had asthma, Total # of units of short-acting beta-2 agonists filled, Whether the patient is single, The maximum percentage of blood eosinophils, The maximum BMI, # of asthma medication orders in the pre-pre-index year, # of asthma diagnoses in the pre-index year, # of diagnoses of asthma with (acute) exacerbation, The proportion having asthma-related hospital encounters out of all patients with asthma in the area, # of medical history diagnosis codes, # of virtual visits, Total # of units of asthma relievers filled, # of major visits for asthma in the pre-pre-index year, The relative change of weight, Whether the patient is Hispanic, Whether the patient is married, Total # of asthma relievers ordered in the pre-pre-index year, Whether the last visits admission type is emergency, Total # of days of gap in asthma controller use, # of ICD- 10 and ICD-9 diagnosis codes, # of days since the last outpatient visit, The average Z-score for weight-for-length, # of days since the last use of asthma medications, Total # of medications filled, Whether the patient has public insurance on the last day, Total # of fills of nebulizer medications, The average # of days per week the patient exercises, The block groups percentage of population 25 and older with college education and no degree, The areas percentage of household income that is between $20,000 and $29,999, # of laboratory tests with abnormal results, The block groups percentage of population 25 and older with less than 9th grade education, # of active problems of diabetes, # of days since taking the last laboratory test, The maximum systolic blood pressure, The average Z-score for length-for-age, Total # of units of asthma relievers filled in the pre-pre-index year, # of days since the last use of short-acting beta-2 agonists, Total # of units of inhaled corticosteroids filled, # of days since the last use of inhaled corticosteroids, # of days since the last virtual visit, # of urgent care visits, # of days since the last use of nebulizer medications, The areas percentage of household income that is between $75,000 and $99,999, Breathing abnormality like dyspnea, The areas percentage of household income that is between $35,000 and $39,999, # of outpatient visits, # of different providers seen in outpatient visits, # of days since the last other type of visit that is not an outpatient visit, an ED visit, a hospitalization, or a virtual visit, # of active problems of obesity, Total # of nebulizer medications ordered, The areas percentage of household income that is between $10,000 and $14,999, The block groups percentage of population 25 and older with 9th- 12th grade education, Total # of different medications ordered, # of years for which the patient had chronic obstructive pulmonary disease, # of patients with asthma of the PCP, # of active problems, # of visits, # of years for which the patient had smoked based on the last record, # of medical history diagnosis codes of asthma, Substance use, The maximum blood eosinophil count, Eczema, Total # of units of asthma medications filled, # of active problems of anxiety/depression, # of active problems of chronic obstructive pulmonary disease, The average peak expiratory flow, Sinusitis, The maximum heart rate, # of active problems of hypertension, Total # of units of asthma controllers filled, The areas percentage of household income that is between $30,000 and $34,999, The average temperature, # of days since the last ED visit, The block groups percentage of population 25 and older with an associates degree, Whether the patient is a female, The areas percentage of non-Hispanic black population, The minimum peak expiratory flow, Total # of medications ordered, Among all patients with asthma of the PCP in the pre-index year, the proportion who had asthma-related hospital encounters in the index year, Upper respiratory tract infection, The areas percentage of household income that is between $40,000 and $44,999, Total copay for asthma medications, The minimum SpO2, # of active problems of asthma in the pre-index year, # of active problems of atherosclerosis, Chronic obstructive pulmonary disease, The areas percentage of non-Hispanic population of two or more races, The areas percentage of non-Hispanic American Indian and Alaska native population, # of days since the last use of systemic corticosteroids, The areas percentage of non-Hispanic other-race population, # of cancelled appointments, Whether the patient was a smoker based on the last record, The areas percentage of household income that is between $50,000 and $59,999, # of different medication prescribers, The areas percentage of Hispanic population of two or more races, The average systolic blood pressure, # of active problems of rhinitis, The average diastolic blood pressure, Whether the patient is white, The average length of stay of an ED visit, The average length of appointment of an outpatient visit with asthma as the primary diagnosis, # of CPT/HCPCS procedure codes, The highest severity of the drug or material allergies the patient had, The block groups percentage of population 25 and older with a high school diploma, The areas percentage of non-Hispanic Asian population, The last ED visits length of stay, # of asthma medication orders, The areas percentage of Hispanic American Indian and Alaska native population, The highest severity of all of the asthma diagnoses, The block groups percentage of population 25 and older with a bachelors degree, Total # of oral steroid prescriptions the PCP ordered for the PCPs patients, The areas percentage of non-Hispanic white population, Whether the patient had any hospitalization, ED visit, or outpatient visit on asthma, The areas percentage of household income that is between $125,000 and $149,999, The areas percentage of Hispanic black population, # of fills of systemic corticosteroids, The maximum respiratory rate, # of active problems of hyperlipidemia, The total length of all of the hospitalizations, # of active problems of asthma in the pre-pre-index year, The areas percentage of household income that is <$10,000, The relative change of BMI, The areas percentage of household income that is between $15,000 and $19,999, Pregnancy, The maximum diastolic blood pressure, The average length of appointment of an outpatient visit, The average # of minutes per week the patient exercises, Whether the patient and the PCP are of the same gender, The areas percentage of Hispanic white population, For the last visit, the time from making the request to the actual visit indicating the requests urgency, Whether the patient used any illicit drug based on the last record, Total # of antibiotic prescriptions the PCP ordered for the PCPs patients, # of hospitalizations, ED visits, and outpatient visits, History of bronchiolitis, Total # of asthma medications filled, # of fills of short-acting beta-2 agonists, # of asthma medications used on the last day, The average asthma control test score, The average # of alcohol drinks the patient had per week across all of the records, The asthma medication ratio, Total # of medications ordered, # of active problems of gastroesophageal reflux disease, The areas percentage of Hispanic other-race population, The last asthma diagnosis severity, Allergic rhinitis, Cataract, The areas percentage of household income that is between $45,000 and $49,999, # of years for which the PCP had practiced at KPSC, Whether the last hospitalization was through the ED, Total # of units of systemic corticosteroids filled, The areas percentage of non-Hispanic native Hawaiian

or other Pacific islander population, Total # of units of asthma reliever medications filled that are neither systemic corticosteroids nor short-acting beta-2 agonists, Total # of opioid prescriptions the PCP ordered for the PCPs patients, Total # of asthma controllers filled, # of allergies of the patient, # of asthma diagnoses in the pre-pre-index year, The minimum post-bronchodilator FEV1/FVC ratio, Indicator of environmental allergy, The areas percentage of Hispanic Asian population, Whether the patient speaks Spanish, # of days of supply of the last asthma controller fill, # of days since the last use of asthma controllers, Whether the most emergent admission type of all of the visits is elective, # of active problems of sleep apnea, The block groups percentage of population 25 and older with a graduate or professional degree, The average # of packs of cigarettes the patient smoked per day across all of the records

§ The mean length of stay of an ED visit, No. of days from the most recent ED visit, No. of primary or principal asthma diagnoses, No. of days from the most recent diagnosis of asthma with (acute) exacerbation or status\nasthmaticus, Whether the patient is black, No. of ED visits, No. of asthma diagnoses, No. of years from the first encounter related to asthma in the data set, No. of nebulizer medication orders, Whether the patient is white, The highest systolic blood pressure, No. of CPT/HCPCS procedure codes, The largest BMI, The most recent ED visits length of stay, Whether the patient is married, Total no. of units of medications ordered, No. of asthma medication orders, Whether nebulizer was used, No. of no shows, Total no. of differing asthma medications ordered, The mean heart rate, No. of diagnoses of asthma with (acute) exacerbation, Whether the patient had any private insurance on the last day, The mean respiratory rate, No. of days from the most recent asthma diagnosis, For the most recent visit, the time to the actual visit after making the request, The mean systolic blood pressure, Total no. of differing medications ordered, Whether the patient has any drug or material allergy, Whether the patient had any public insurance on the last day, The lowest SpO2, No. of active problems, Whether the most recent visit is an ED visit, The highest temperature, No. of laboratory tests, No. of asthma controller orders, No. of visits, Total no. of short-acting beta-2 agonists ordered, The largest blood eosinophil count, No. of asthma medication orders, Total no. of asthma relievers ordered that are neither short-acting beta-2 agonists nor\nsystemic corticosteroids, The mean temperature, Total no. of inhaled corticosteroids ordered, No. of days from the most recent outpatient visit on asthma, The severity of the most recent asthma diagnosis, Total no. of refills permitted for short-acting beta-2 agonists, No. of differing providers the patient saw in outpatient visits, Age, No. of outpatient visits to the patients PCP, No. of laboratory tests having abnormal results, Total no. of systemic corticosteroids ordered, Whether the most recent asthma diagnosis is a primary or principal one, Whether the patient is single, The day of the week when the most recent ED visit began, The relative change of BMI, The mean length of a hospitalization, No. of days from the most recent ED visit on asthma, No. of active problems of asthma, Total no. of differing nebulizer medications ordered, Total no. of differing asthma relievers ordered, Total no. of refills permitted for asthma controllers, Whether the patient has any mental disorder, The relative change of weight, Whether the most recent visits admission type is emergency, Whether the patient was a smoker according to the most recent record, Whether the most recent hospitalization came from the ED, The severity of the most severe asthma diagnosis, No. of outpatient visits, The highest diastolic blood pressure
