# Supplementary material for: Machine learning for prediction of asthma exacerbations among asthmatic patients: a systematic review and meta-analysis
Source: BMC Pulm Med. 2023 Jul 28;23:278. doi: 10.1186/s12890-023-02570-w (PMC10386701; doi:10.1186/s12890-023-02570-w)
Supplement: Supplementary file 8 — Additional file 8: The overall pooled positive likelihood ratio, negative likelihood ratio, and diagnostic odds ratio of 23 machine learning prediction models. [file 12890_2023_2570_MOESM8_ESM.pdf]

Additional file 8. The overall pooled positive likelihood ratio, negative likelihood ratio, and diagnostic odds ratio of 23 machine learning prediction models

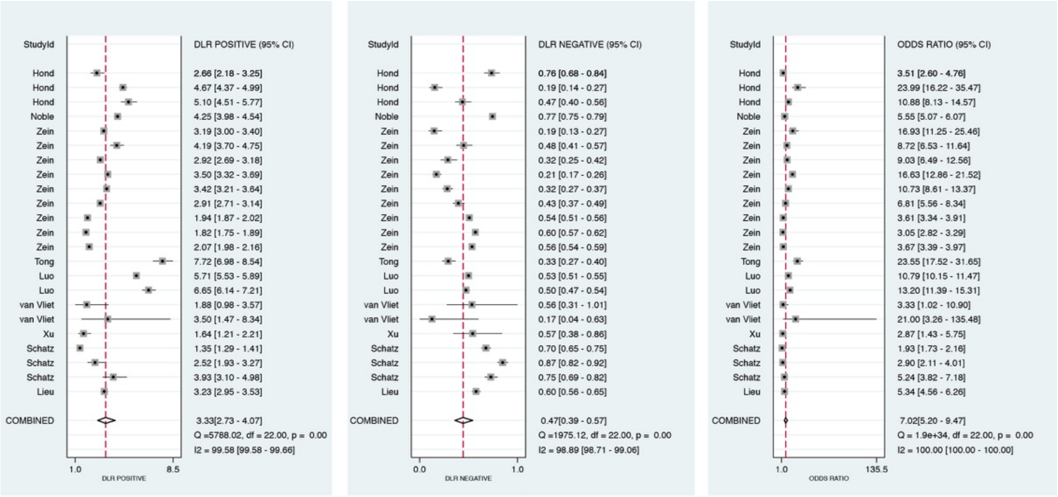

a b c
