# Supplementary material for: Machine learning for prediction of asthma exacerbations among asthmatic patients: a systematic review and meta-analysis
Source: BMC Pulm Med. 2023 Jul 28;23:278. doi: 10.1186/s12890-023-02570-w (PMC10386701; doi:10.1186/s12890-023-02570-w)
Supplement: Supplementary file 9 — Additional file 9: Forest plots of performance measures in subgroup analysis. [file 12890_2023_2570_MOESM9_ESM.pdf]

Additional file 9. Forest plots of performance measures in subgroup analysis

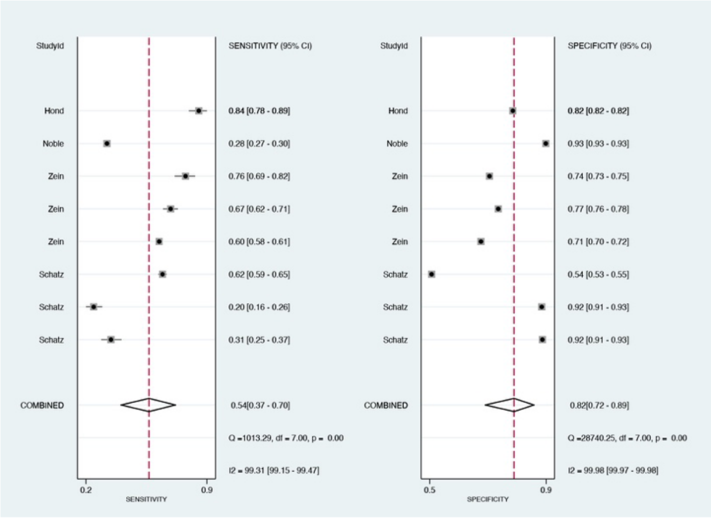

a

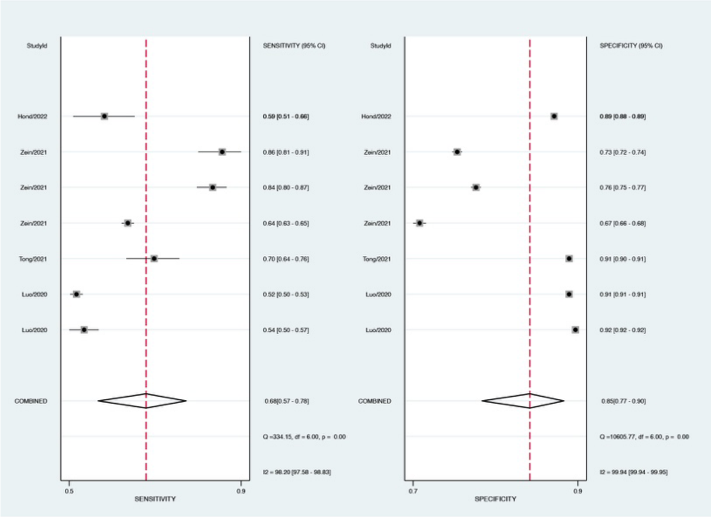

b

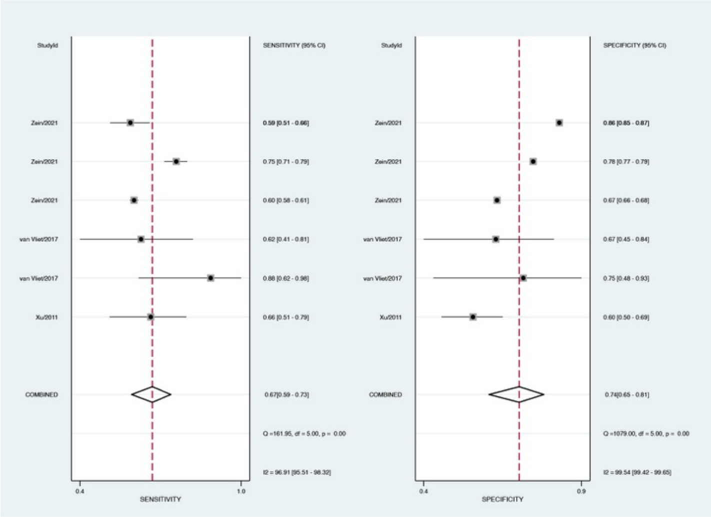

c

The pooled sensitivity and specificity of machine learning prediction models stratified by logistic regression(a), boosting(b), and random forest(c) method

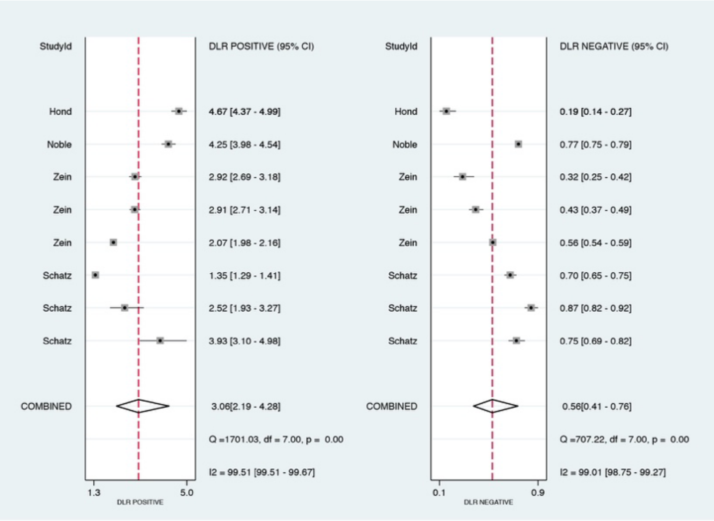

a

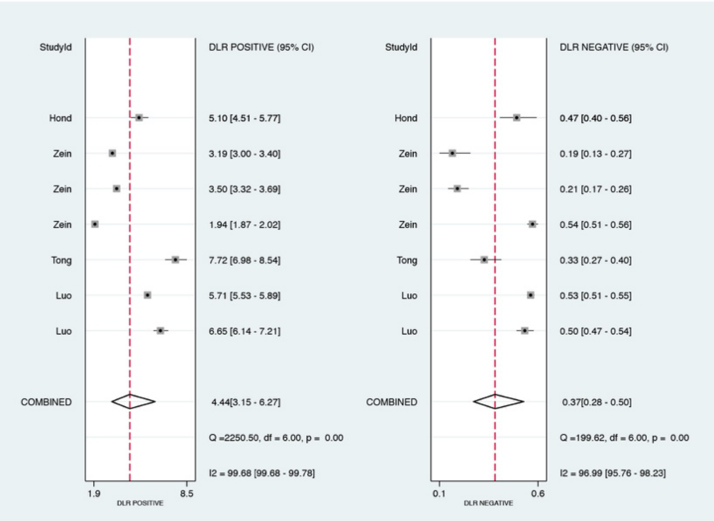

b

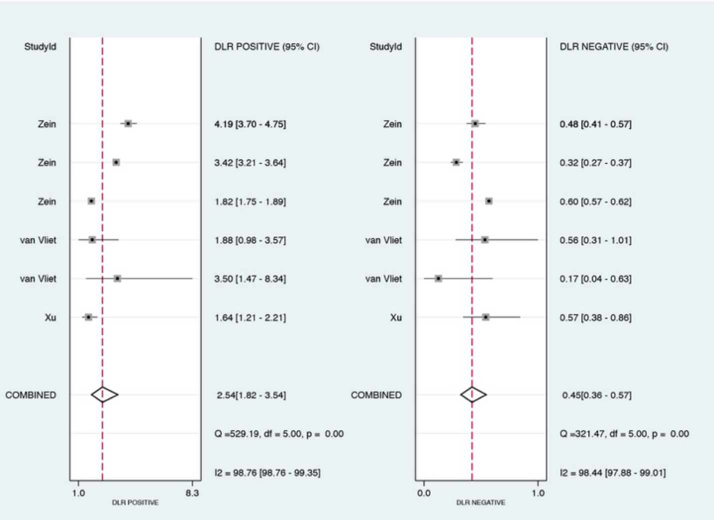

c

The pooled positive likelihood ratio and negative likelihood ratio of machine learning prediction models stratified by logistic regression(a), boosting(b), and random forest(c) methods.

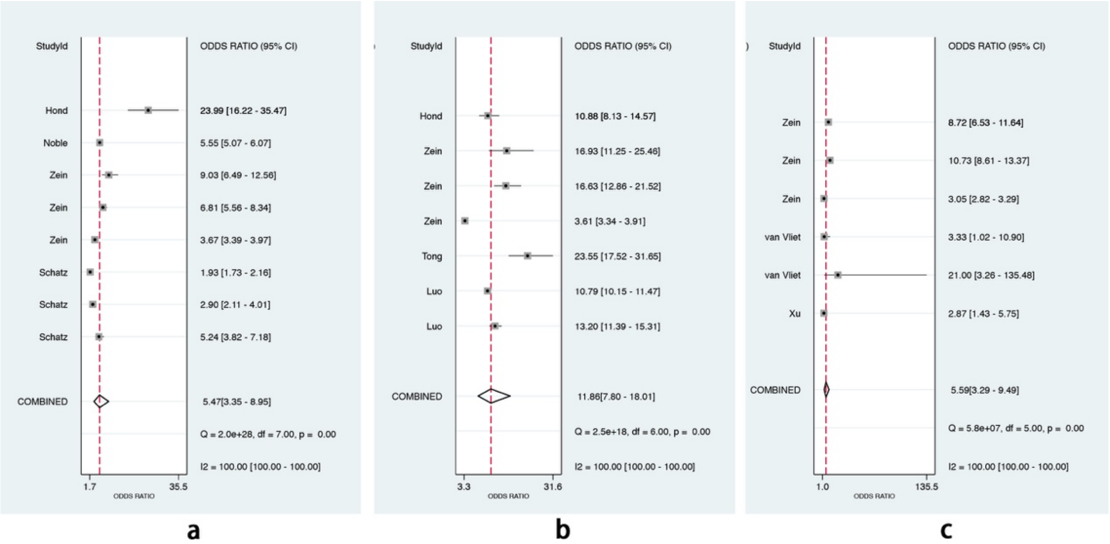

The pooled diagnostic odds ratio of machine learning prediction models stratified by logistic regression(a), boosting(b), and random forest(c) methods.

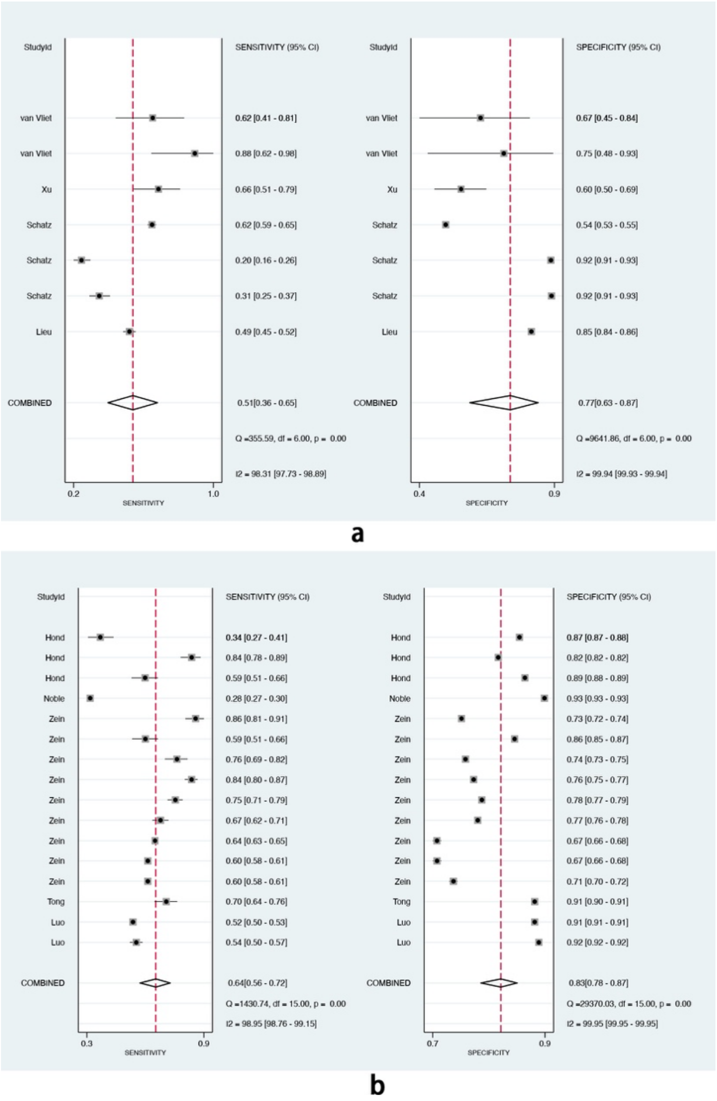

The pooled sensitivity and specificity of machine learning prediction models stratified by different sample sizes. (a) Sample size < 10000. (b) Sample size > 10000.

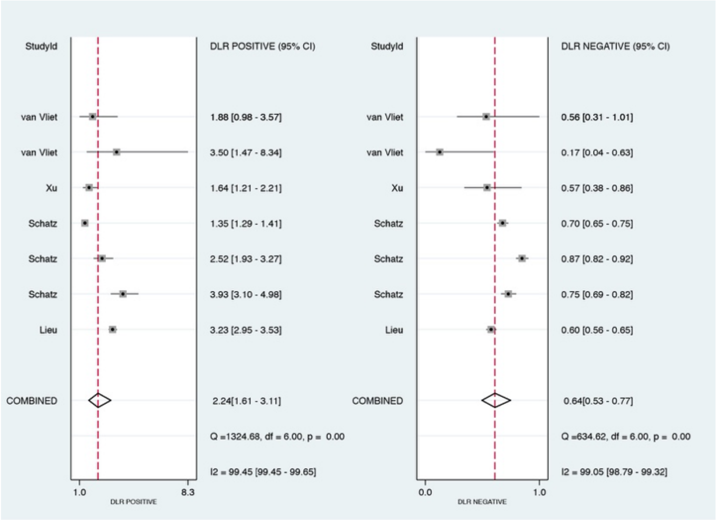

a

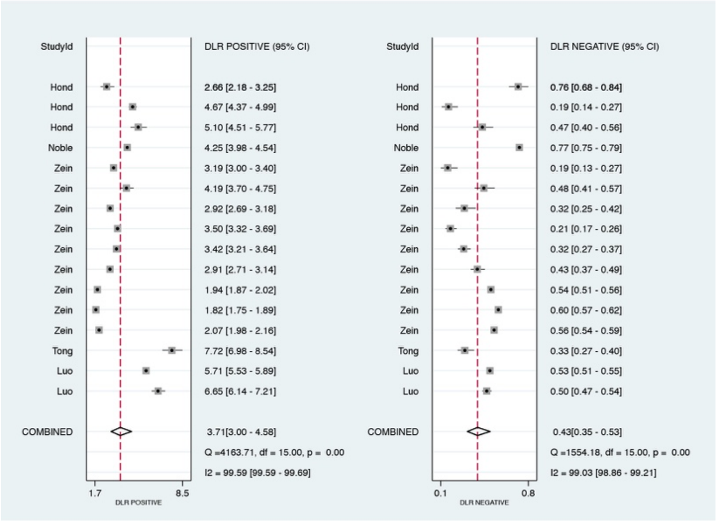

b

The pooled positive likelihood ratio and negative likelihood ratio of machine learning prediction models stratified by different sample sizes. (a) Sample size < 10000. (b) Sample size > 10000.

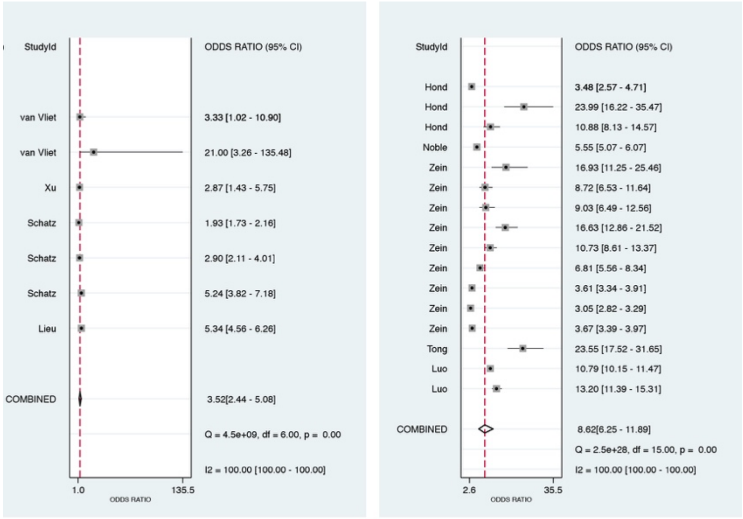

a **b**

The diagnostic odds ratio of machine learning prediction models stratified by different sample sizes. (a) Sample size < 10000. (b) Sample size > 10000.

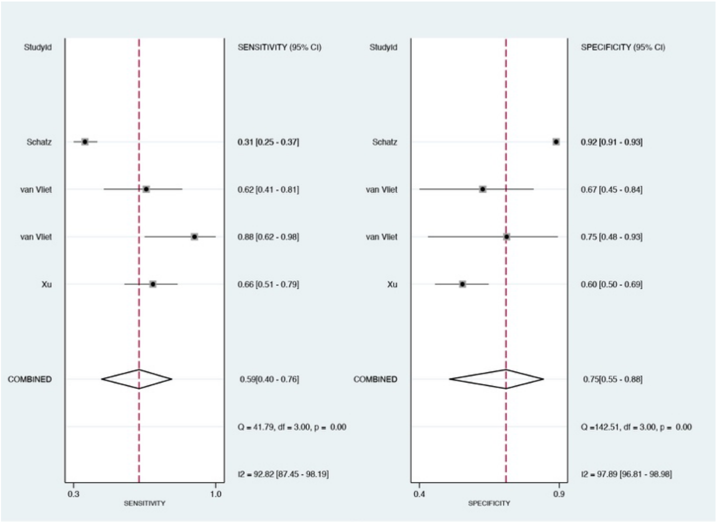

a

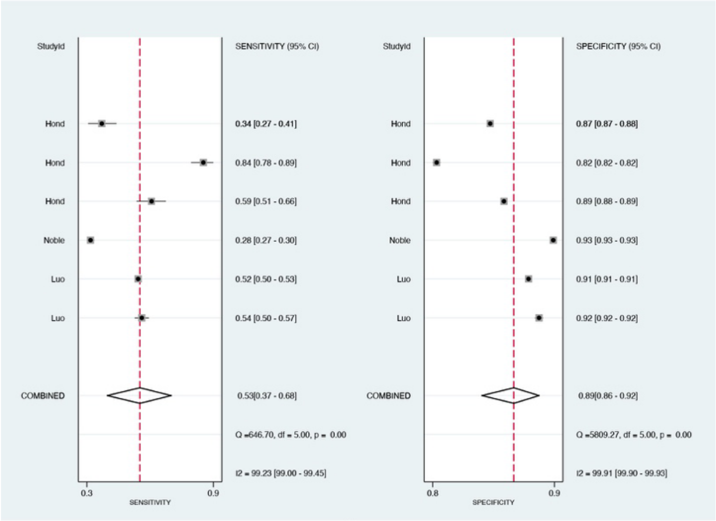

b

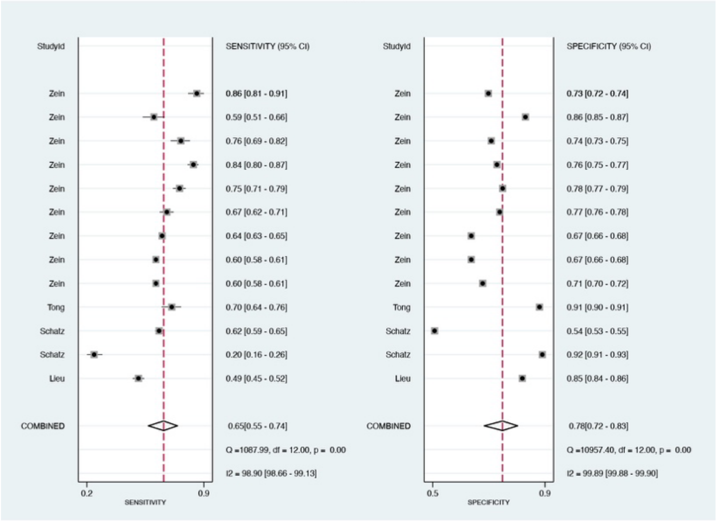

c

The pooled sensitivity and specificity of machine learning prediction models stratified by different age groups: (a) Children. (b) Children and adults. (c) Adults.

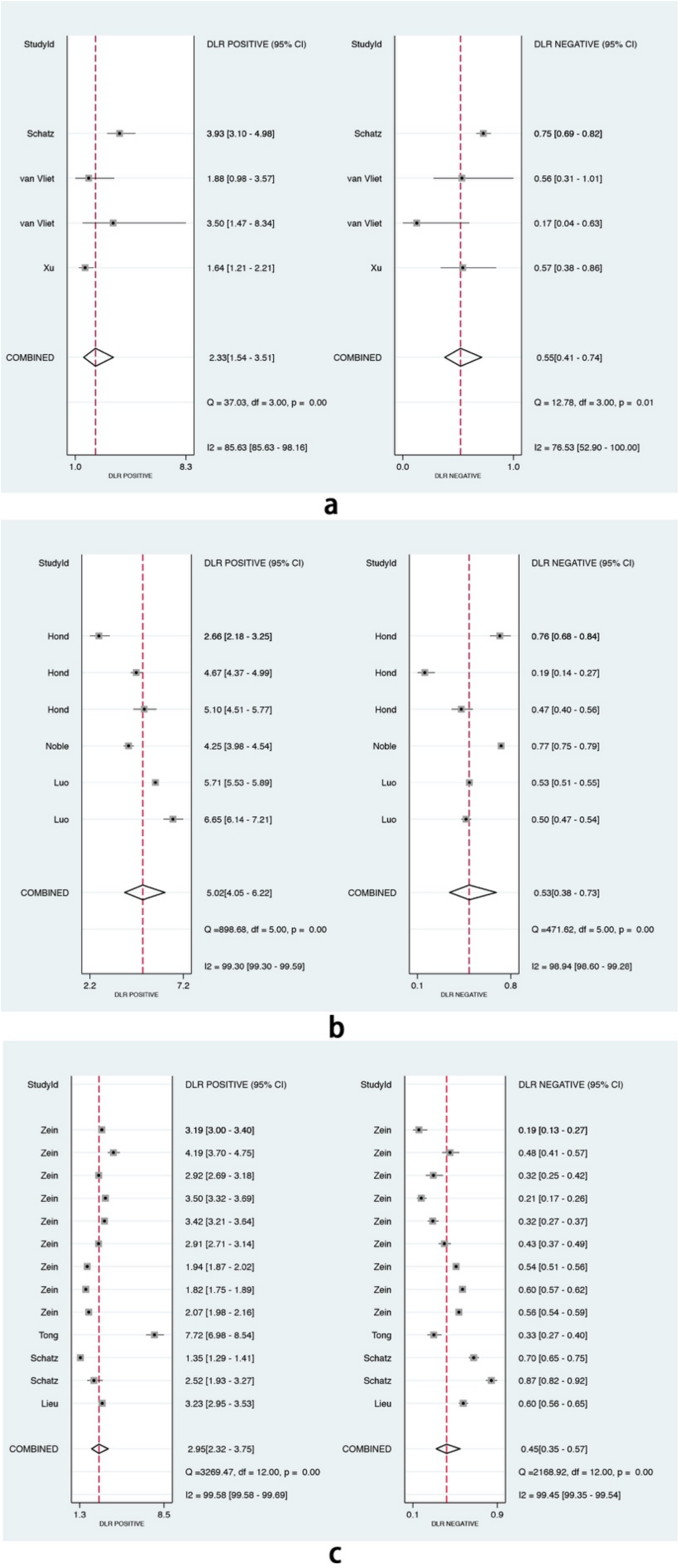

The pooled positive likelihood ratio and negative likelihood ratio of machine learning prediction models stratified by different age groups: (a) Children. (b) Children and adults. (c) Adults.

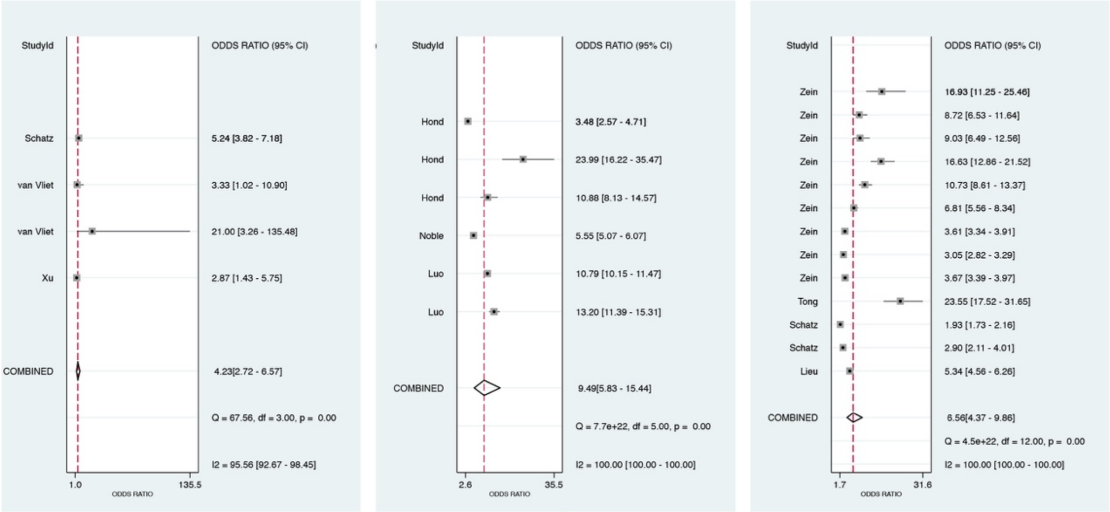

a **b** **c**

The diagnostic odds ratio of machine learning prediction models stratified by different age groups: (a) Children. (b) Children and adults. (c) Adults.

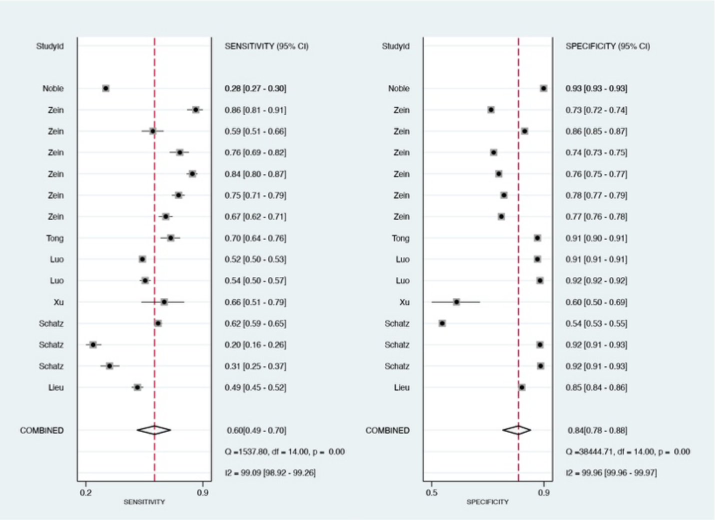

a

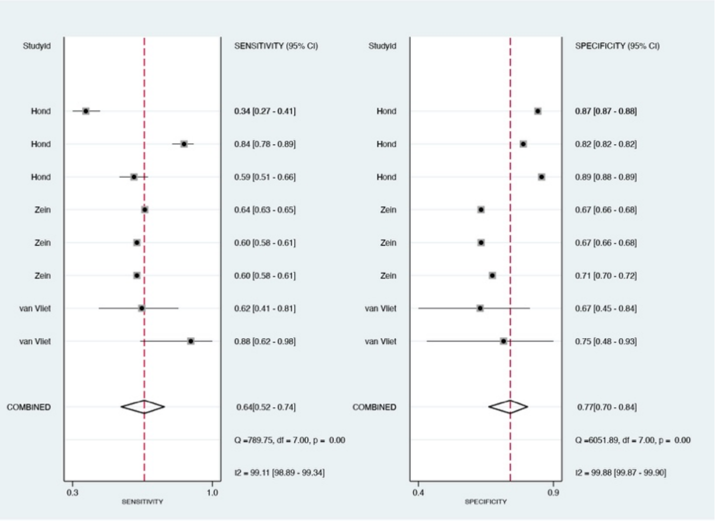

b

The pooled sensitivity and specificity of machine learning prediction models stratified by different outcome definitions: (a) Emergency department visits/ hospitalization for

asthma. (b) Asthma exacerbation definition in accordance with ATS/ERS statement.

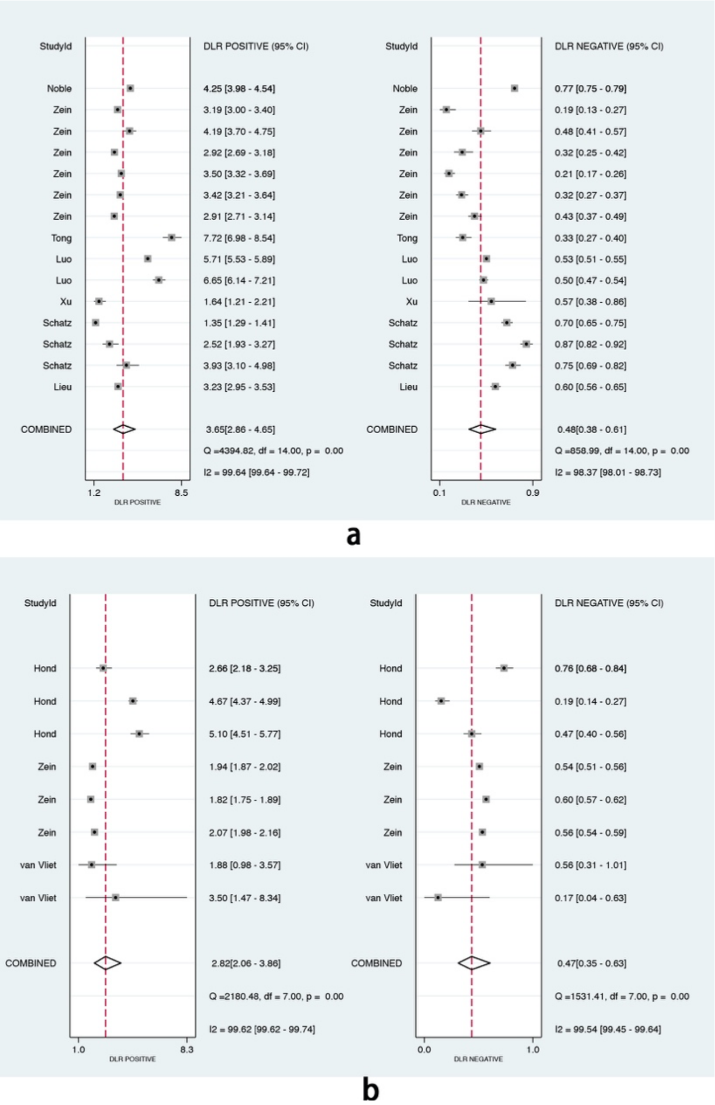

The pooled positive likelihood ratio and negative likelihood ratio of machine learning prediction models stratified by different outcome definitions: (a) Emergency department visits/ hospitalization for asthma. (b) Asthma exacerbation definition in accordance with ATS/ERS statement.

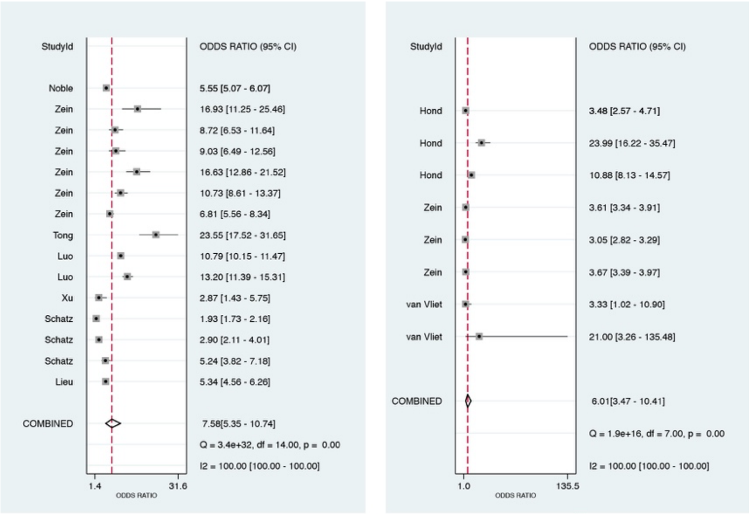

a **b**

The diagnostic odds ratio of machine learning prediction models stratified by different outcome definitions: (a) Emergency department visits/ hospitalization for asthma. (b) Asthma exacerbation definition in accordance with ATS/ERS statement.
