# Supplementary material for: Machine learning for prediction of asthma exacerbations among asthmatic patients: a systematic review and meta-analysis
Source: BMC Pulm Med. 2023 Jul 28;23:278. doi: 10.1186/s12890-023-02570-w (PMC10386701; doi:10.1186/s12890-023-02570-w)
Supplement: Supplementary file 10 — Additional file 10: The influence of each model for the outcome of meta-analysis [file 12890_2023_2570_MOESM10_ESM.pdf]

Additional file 10. The influence of each model for the outcome of meta-analysis

Study	Pooled AUROC (95% CI)	Pooled sensitivity (95% CI)	Pooled specificity (95% CI)	Pooled PLR (95% CI)	Pooled NLR (95% CI)	Pooled DOR (95% CI)
Lieu 1999	0.80(0.76-0.83)	0.63(0.55-0.70)	0.81(0.75-0.85)	3.3(2.7-4.1)	0.45(0.37-0.55)	7(5-10)
Schatz 2004	0.79(0.76-0.83)	0.65(0.57-0.71)	0.80(0.75-0.85)	3.3(2.6-4.0)	0.44(0.37-0.53)	7(5-10)
Schatz 2006	0.81(0.77-0.84)	0.63(0.55-0.71)	0.82(0.77-0.86)	3.5(2.9-4.2)	0.45(0.37-0.55)	8(6-10)
Xu 2011	0.80(0.77-0.83)	0.63(0.54-0.70)	0.82(0.77-0.86)	3.4(2.8-4.2)	0.46(0.38-0.55)	7(5-10)
Van Vliet 2017	0.79(0.75-0.82)	0.62(0.54-0.69)	0.81(0.76-0.85)	3.3(2.7-4.1)	0.47(0.39-0.56)	7(5-10)
Van Vliet 2017	0.80(0.76-0.83)	0.63(0.55-0.70)	0.81(0.76-0.86)	3.4(2.7-4.1)	0.46(0.38-0.55)	7(5-10)
Luo 2020	0.79(0.76-0.83)	0.63(0.55-0.71)	0.80(0.75-0.85)	3.3(2.6-3.9)	0.46(0.38-0.55)	7(5-10)
Luo 2020	0.79(0.76-0.83)	0.63(0.55-0.71)	0.80(0.75-0.85)	3.3(2.6-4.0)	0.46(0.37-0.55)	7(5-10)
Tong 2021	0.79(0.75-0.82)	0.62(0.54-0.70)	0.80(0.75-0.85)	3.3(2.6-3.9)	0.47(0.39-0.56)	7(5-9)
Zein 2021	0.80(0.76-0.83)	0.63(0.55-0.71)	0.81(0.76-0.86)	3.4(2.7-4.2)	0.45(0.37-0.55)	7(5-10)
Zein 2021	0.80(0.76-0.83)	0.63(0.55-0.71)	0.82(0.76-0.86)	3.4(2.8-4.2)	0.45(0.37-0.55)	8(6-10)
Zein 2021	0.80(0.76-0.83)	0.63(0.55-0.70)	0.82(0.76-0.86)	3.4(2.8-4.2)	0.46(0.38-0.55)	7(5-10)
Zein 2021	0.80(0.76-0.83)	0.63(0.54-0.70)	0.81(0.76-0.85)	3.3(2.7-4.1)	0.46(0.38-0.56)	7(5-10)
Zein 2021	0.79(0.75-0.83)	0.62(0.54-0.70)	0.81(0.76-0.85)	3.3(2.6-4.1)	0.47(0.39-0.56)	7(5-10)
Zein 2021	0.79(0.75-0.82)	0.61(0.54-0.69)	0.81(0.76-0.85)	3.3(2.6-4.0)	0.47(0.40-0.57)	7(5-9)
Zein 2021	0.79(0.76-0.83)	0.62(0.54-0.70)	0.81(0.76-0.86)	3.3(2.7-4.1)	0.47(0.39-0.56)	7(5-10)
Zein 2021	0.79(0.76-0.83)	0.63(0.55-0.71)	0.81(0.75-0.85)	3.3(2.6-4.0)	0.46(0.38-0.55)	7(5-10)
Zein 2021	0.79(0.75-0.82)	0.61(0.54-0.69)	0.81(0.76-0.86)	3.3(2.6-4.1)	0.48(0.40-0.57)	7(5-9)
Noble 2021	0.80(0.76-0.83)	0.64(0.57-0.71)	0.80(0.75-0.84)	3.2(2.6-4.0)	0.44(0.37-0.53)	7(5-10)
Hond 2022	0.79(0.76-0.83)	0.63(0.55-0.71)	0.80(0.75-0.85)	3.3(2.6-4.0)	0.46(0.38-0.56)	7(5-10)
Hond 2022	0.79(0.75-0.82)	0.62(0.54-0.69)	0.81(0.76-0.85)	3.2(2.6-4.0)	0.48(0.40-0.56)	7(5-9)
Hond 2022	0.80(0.76-0.83)	0.64(0.56-0.71)	0.81(0.75-0.85)	3.3(2.7-4.1)	0.44(0.37-0.53)	7(5-10)
